# The genome of the warm-season turfgrass African bermudagrass (*Cynodon transvaalensis*)

**DOI:** 10.1038/s41438-021-00519-w

**Published:** 2021-05-01

**Authors:** Fengchao Cui, Geli Taier, Manli Li, Xiaoxia Dai, Nan Hang, Xunzhong Zhang, Xiangfeng Wang, Kehua Wang

**Affiliations:** 1 Department of Turfgrass Science and Engineering, College of Grassland Science and Technology, China Agricultural University, Beijing, 100193 China; 2 Department of Breeding and Seed Science, College of Grassland Science and Technology, China Agricultural University, Beijing, 100193 China; 3 School of Plant and Environmental Sciences, Virginia Polytechnic Institute and State University, Blacksburg, VA 24061 USA; 4 National Maize Improvement Center, College of Agronomy and Biotechnology, China Agricultural University, Beijing, 100913 China

**Keywords:** Comparative genomics, Plant evolution

## Abstract

*Cynodon* species can be used for multiple purposes and have high economic and ecological significance. However, the genetic basis of the favorable agronomic traits of *Cynodon* species is poorly understood, partially due to the limited availability of genomic resources. In this study, we report a chromosome-scale genome assembly of a diploid *Cynodon* species, C. transvaalensis, obtained by combining Illumina and Nanopore sequencing, BioNano, and Hi-C. The assembly contains 282 scaffolds (~423.42 Mb, N50 = 5.37 Mb), which cover ~93.2% of the estimated genome of C. transvaalensis (~454.4 Mb). Furthermore, 90.48% of the scaffolds (~383.08 Mb) were anchored to nine pseudomolecules, of which the largest was 60.78 Mb in length. Evolutionary analysis along with transcriptome comparison provided a preliminary genomic basis for the adaptation of this species to tropical and/or subtropical climates, typically with dry summers. The genomic resources generated in this study will not only facilitate evolutionary studies of the Chloridoideae subfamily, in particular, the Cynodonteae tribe, but also facilitate functional genomic research and genetic breeding in *Cynodon* species for new leading turfgrass cultivars in the future.

## Introduction

Bermudagrasses (*Cynodon* spp.) are warm-season (C_4_) perennial grass species that originate primarily from open areas in southeastern Africa^1^. Following the early diversification associated with the grazing of herbivorous African hoofed animals, their secondary/postancestral centers of origin/diversification occurred in multiple locations, including in South Africa, India, Australia, Afghanistan, and China^2,3^. The *Cynodon* genus (*Cynodon* Rich.) is classified in the *Cynodonteae* tribe, Chloridoideae subfamily, and Poaceae family, with limited diversification of ~9–11 species, including *Cynodon aethiopicus* Clayton & Harlan, *C. barberi* Rang. & Tadul., *C. coursii* A. Camus, *C. dactylon* (L.) Pers., *C. incompletus* Nees, *C.* *×* *magennisii* Hurcombe, *C. nlemfuensis* Vanderyst, *C. parviglumis* Ohwi, *C. plectostachyus* (K. Schum.) Pilg., C. *radiatus* Roth, and *C. transvaalensis* Burtt Davy^4–7^ (The Plant List, www.theplantlist.org, 2012). Bermudagrasses are widespread and troublesome weeds, but more importantly, owing to a number of important characteristics of *Cynodon* species, they can be used for multiple purposes with enormous economic and ecological significance^8,9^. In particular, the good tolerance to abiotic stresses (i.e., traffic, heat, salinity, and drought), ability to grow in nearly all types of soil conditions, rapid recovery potential, low growing nature and aggressive sod-forming growth habit with rhizomes and solons all make bermudagrasses the most used warm-season turfgrasses worldwide in the turf industry, and these grasses are ideally suited for any kind of application, including for ground covers, lawns, sports fields, golf course tees, fairways, and putting greens^6,10^. They are also important forages in the southern United States^11^ and have attracted research interest as biofuel grasses^12^, phytoremediation plants for soil reclamation^13,14^, and medicinal plants for their pharmacognostic properties (e.g., as anti-inflammatory, diuretic, antiemetic, antidiabetic, and blood-purifying agents) in Asia and Australia^15,16^. However, the genetic basis of the favorable agronomic traits of *Cynodon* species is poorly understood, partially due to the limited availability of genetic and genomic resources.

The genus *Cynodon* belongs to the “PACC” clade that contains the subfamilies Panicoideae, Arundinoideae, Centothecoideae, and Chloridoideae^17^ or the current “PACMAD” clade, which regroups six subfamilies, namely, Panicoideae, Aristidoideae, Chloridoideae, Micrairoideae, Arundinoideae, and Danthonioideae^18,19^. The “PACMAD” lineage is the only grass group within which the C_4_ photosynthesis pathway has evolved, and Panicoideae and Chloridoideae are the two major groups in the clade, with ~3600 and 1600 species, respectively^7,20,21^. Compared to the Panicoideae subfamily, which includes some important agricultural crops that have been extensively studied, such as sorghum, maize, sugarcane, foxtail millet, and switchgrass, the Chloridoideae subfamily largely lags behind in terms of scientific exploration, particularly at the whole-genome level. Moreover, among the four most important genera within Chloridoideae, namely, *Cynodon*, *Eleusine*, *Zoysia*, and *Eragrostris*, *Cynodon* currently lags behind the other three in terms of genomics and molecular breeding applications^9,22^. For instance, several reference genomes have been reported for each of the three genera, such as two for *Eleusine* (i.e., finger millet, *E. coracana*; goosegrass, *E. indica*)^23,24^, three for *Zoysia* (i.e., *Z. japonica*, *Z. matrella*, and *Z. pacifica*)^25^, and three for *Eragrostris* (i.e., *E. nindensis*; weeping love grass, *E. curvula*; tef, *E. tef*)^26,27^, but so far, there is no reference genome available for the *Cynodon* genus, which significantly limits our understanding of its evolutionary history and our ability to exploit the full genetic potential of these species for molecular breeding of superior cultivars, particularly in the face of global climate change and worldwide water shortage.

*Cynodon* plant species can range from diploid to hexaploid and have 2n chromosome numbers from 18 to 54^1,4,28,29^. Among all the *Cynodon* species, the two with the highest scientific, industrial and economic importance are C. dactylon and C. transvaalensis, namely, common bermudagrass and African bermudagrass, respectively. Common bermudagrass, also known as dog’s tooth grass, Bahama grass, devil’s grass, couch grass, Indian doab, wiregrass, and scutch grass, is a cosmopolitan grass that is native to most of the eastern hemisphere and is now found throughout the tropical, subtropical, and warm-temperate climatic regions of the world^30^. In contrast, African bermudagrass is more confined; this species is native to only South Africa and has now been introduced to Madagascar, Central and Northwest Africa, Greece, Iran, Southeast Australia, and part of the United States, with relatively limited dispersal and naturalization^30,31^. Common bermudagrass is usually tetraploid with 36 chromosomes (2*n* = 4*x* = 36), while African bermudagrass is diploid with 18 chromosomes (2*n* = 2*x* = 18)^2,28^. A number of leading varieties/cultivars used for turfgrass are sterile triploid hybrids from natural (*C.* *×* *magennisii*) or human-made crosses between common bermudagrass and African bermudagrass, such as Tifgreen, Tifway, Tifdwrf, MS Supreme, Champion, and MiniVerde^10^.

Here, we report a chromosome-scale genome assembly of C. transvaalensis obtained by combining Illumina and Nanopore sequencing, BioNano, and Hi-C. The assembly contains 282 scaffolds (~423.42 Mb, N50 = 5.37 Mb), which cover ~93.2% of the estimated genome of C. transvaalensis (~454.4 Mb). We annotated 28,444 genes in C. *transvaalensis*, which is comparable to the gene number of another Cynodonteae tribe species, *Oropetium thomaeum* (28,446–28,835 genes, genome size of ~236–245 Mb)^32,33^. The expanded genes of C. transvaalensis are primarily involved in disease resistance and abiotic stress responses. Transcriptomic analysis of the plants under various abiotic stresses (i.e., high temperature, cold, drought, and salinity) provided further information for understanding the mechanism underlying its responses/adaptation to changing environments. As the first de novo genome assembly of *Cynodon* species in the genus, the genome resources generated in this study will not only facilitate evolutionary studies of the Chloridoideae subfamily, in particular, the Cynodonteae tribe, but also facilitate functional genomic research and genetic breeding in *Cynodon* species for new leading turfgrass cultivars in the future.

## Results

### Genome sequencing, assembly, and evaluation

**Fig. 1:**
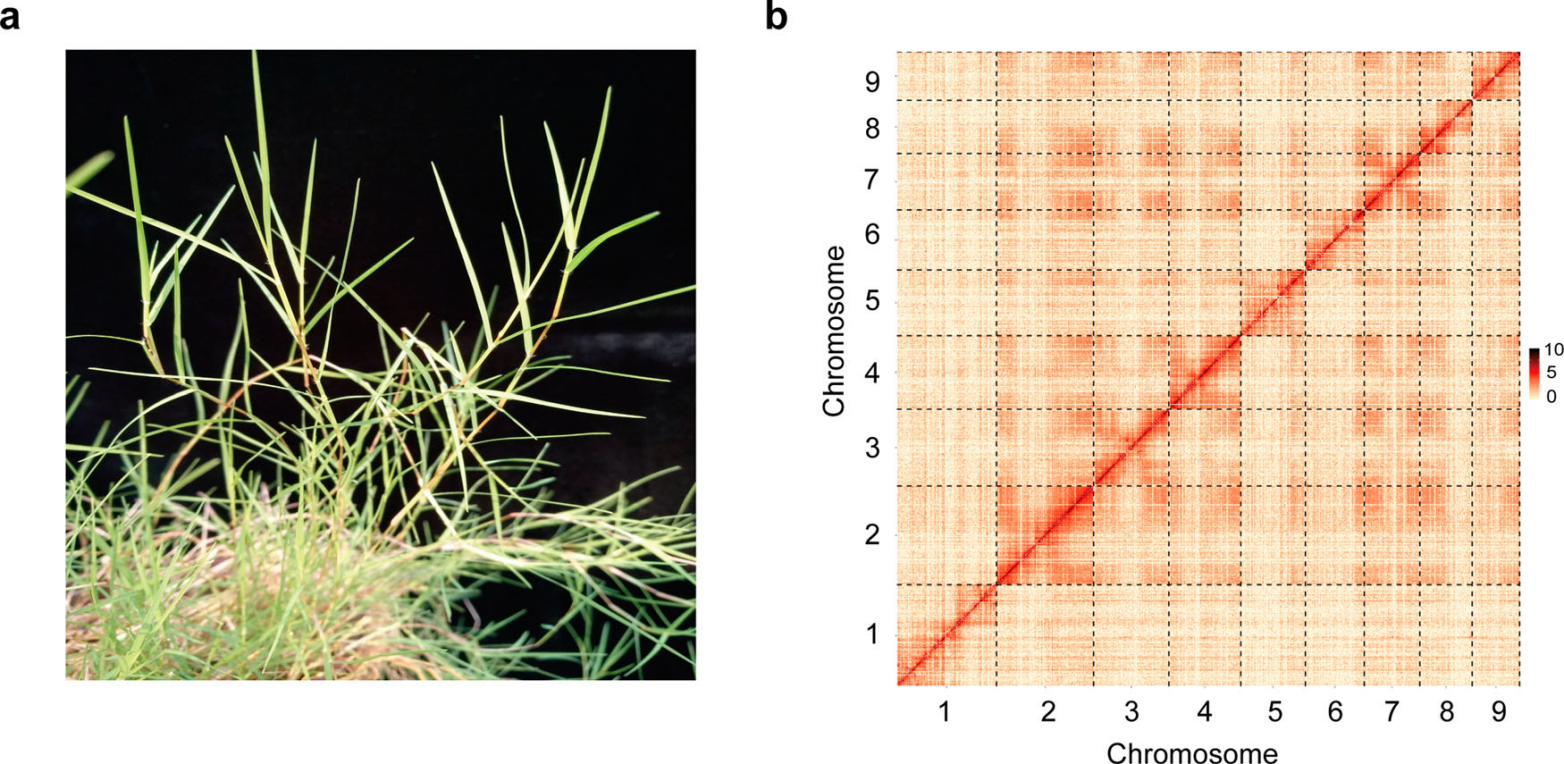
Plant morphology and Hi-C-assisted genome assembly of *C. transvaalensis*. **a** Phenotype of the sequenced *C. transvaalensis* plant. **b** Hi-C interaction heatmap showing 100-kb resolution superscaffolds

In this study, we used *C. transvaalensis* (Fig. 1a) for whole-genome sequencing^34^. Based on Illumina data (~18.8 Gb), *k*-mer frequency analysis revealed that the genome size was ~454.4 Mb (Supplementary Fig. 1), similar to the size determined by flow cytometry (~444.3 Mb) (Supplementary Fig. 2). The heterozygosity was also evaluated to be 1.40%, and the high heterozygosity rate led to an obvious secondary peak at the 1/2 position from the main peak (Supplementary Fig. 1).

In order to overcome the difficulty of assembly with high levels of heterozygosity, a series of technologies, including Illumina sequencing, Nanopore sequencing, Bionano, and Hi-C, were utilized to assemble the genome, and the sequencing and assembly workflow is shown in Supplementary Fig. 3. In this process, a total of 80.13-Gb Nanopore long reads (~176.3× coverage of the genome, N50 = 28 kb) were de novo assembled into contigs, and the contigs were polished by using Illumina short reads (~41.4× coverage of the genome, Supplementary Table 1). The assembled genome (~420.45 Mb) contained 327 contigs, in which the contig N50 and the longest contig were 2.58 and 7.99 Mb, respectively (Table 1). Subsequently, ~52.6 Gb of Bionano optical maps (~115.8× coverage of the genome, N50 = 3.39 Mb) were applied to integrate contig sequences into scaffolds and to improve the assembly accuracy (Supplementary Fig. 4), and then, gap sequences longer than 200 kb were deleted (Supplementary Table 2). The remaining 282 scaffolds (~423.42 Mb) accounted for 95.30% of the genome determined by flow cytometry, and the scaffold N50 and the longest scaffold were 5.37 and 35.13 Mb, respectively. Hi-C data (~77.3 Gb, ~170.10× coverage of the genome) were then used to correct, sort, and orient the scaffolds into chromosome-level superscaffolds (pseudomolecules) (Fig. 1b). In total, 90.48% of the scaffolds (~383.08 Mb) were anchored to nine pseudomolecules, of which the longest was 60.78 Mb. The average GC content of the assembled genome was 43.4%, which was close to that of rice (43.6%)^35^ and lower than that of zoysiagrass (44.1%)^25^. The assembly information was summarized in Table 1.

**Table 1:** Summary statistics for the *C. transvaalensis* genome

Estimated genome size	444.30 Mb
Total length of assembly	423.42 Mb
Number of contigs	327
Contig N50	2582,667 bp
Largest contig	7992,975 bp
Number of scaffolds	282
Scaffold N50	5371,758 bp
Largest scaffold	35,132,054 bp
Chromosome length	383.08 Mb
GC content of the genome	43.4%
Number of gene models	28,444
Mean gene length	5350 bp
Mean coding sequence length	1280 bp
Total size of transposable elements	128.14 Mb

To evaluate the quality and integrity of the assembly, Illumina reads were aligned to the genome, and the mapping ratio was 97.62%. In addition, the completeness of the genetic space was assessed by using 1375 Benchmarking Universal Single-Copy Orthologs (BUSCO) genes from Embryophyta^36^, and the results indicated that 97.9% of the genes could be annotated and 97% of them were complete (Supplementary Table 3).

### Gene annotation

In the assembled genome, repetitive sequences and transposable elements (TEs) accounted for 38.11% and 33.45% of the total sequence, respectively. Among the TEs, long terminal repeat (LTR) retrotransposons were predominant, accounting for 22.14% of the genome. The percentages of *Gypsy* and *Copia* LTR retrotransposons were 14.85% and 2.15% (Supplementary Table 4), respectively. Moreover, long interspersed nuclear elements, short interspersed nuclear elements, and DNA transposons covered 2.9%, 0.01% and 7.49% of the genome, respectively. We also identified 117,768 simple sequence repeats (SSRs) for the assembled genome (Supplementary Table 5).

**Fig. 2:**
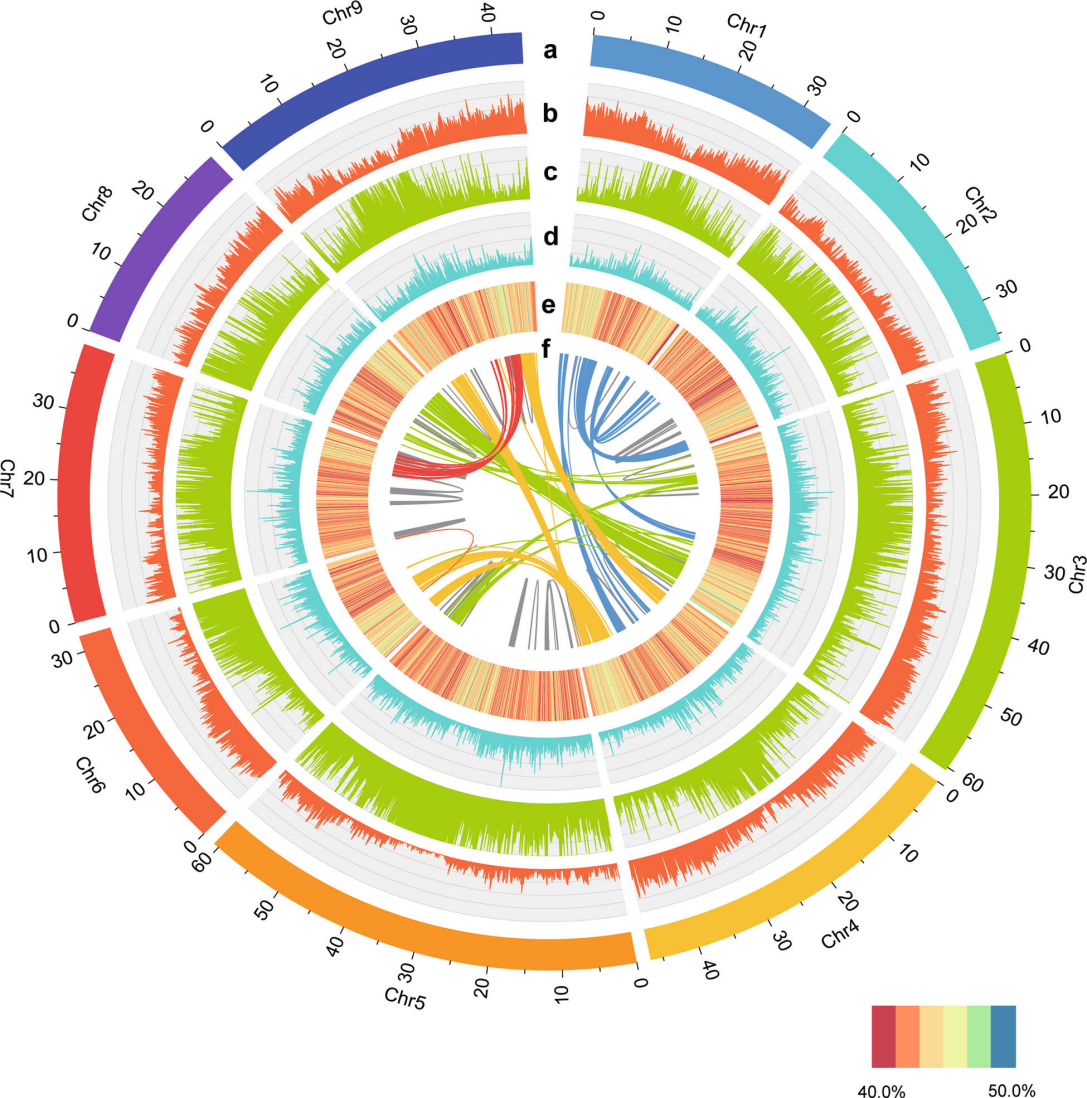
Features of the *C. transvaalensis* genome. (a) Length of each pseudomolecule. (b) Density of genes. (c) Distribution of *Gypsy*-type retrotransposons. (d) Distribution of *Copia*-type retrotransposons. (e) Heatmap of the GC content. (f) Whole-genome duplication (WGD) events shown by syntenic relationships, with blocks containing more than ten paralogous gene pairs

By combining the transcriptome, homolog and ab initio prediction strategies (Supplementary Table 6), a total of 28,444 high-confidence gene models were obtained after removing gene models containing TEs, which were unevenly distributed on nine pseudomolecules. The average length of the genes was 5350 bp, and each gene contained 5.3 exons. The average CDS, exon, and intron lengths were 1281, 241, and 944 bp, respectively. The gene density, GC content, and *Gypsy* and *Copia* distributions on each pseudomolecule are shown in a circular diagram (Fig. 2). We also compared the *C. transvaalensis* genome with five related C_4_ species (*Zoysia japonica*, *Zea mays*, *Sorghum bicolor*, *Setaria italica*, and *Setaria viridis*). Among them, the maize genome was the largest (~2.1 Gb), being ~5.5 times larger than the *C. transvaalensis* genome (Supplementary Fig. 5a). Zoysiagrass had the largest number of genes (49,103), while *C. transvaalensis* had the fewest genes (28,444). The other four species had similar gene numbers (Supplementary Fig. 5b). We found that the distribution of CDSs and gene lengths of the six species showed similar patterns (Supplementary Fig. 5c, d). Gene functions were also validated by searching the predicted gene models in the databases (KEGG, GO, KOG, Swiss-Prot, Nr). In total, 27,146 genes (95.44%) could be annotated, 75.38% and 95.20% of which were found in Swiss-Prot and Nr (Supplementary Fig. 6a), respectively, and 4458 gene models could be annotated across the five databases (Supplementary Fig. 6b). Moreover, 0.06% of the genome was annotated as noncoding RNAs, which included 803 miRNAs, 760 tRNAs, and 62 rRNAs (Supplementary Table 7).

### Gene family and evolution analysis

**Fig. 3:**
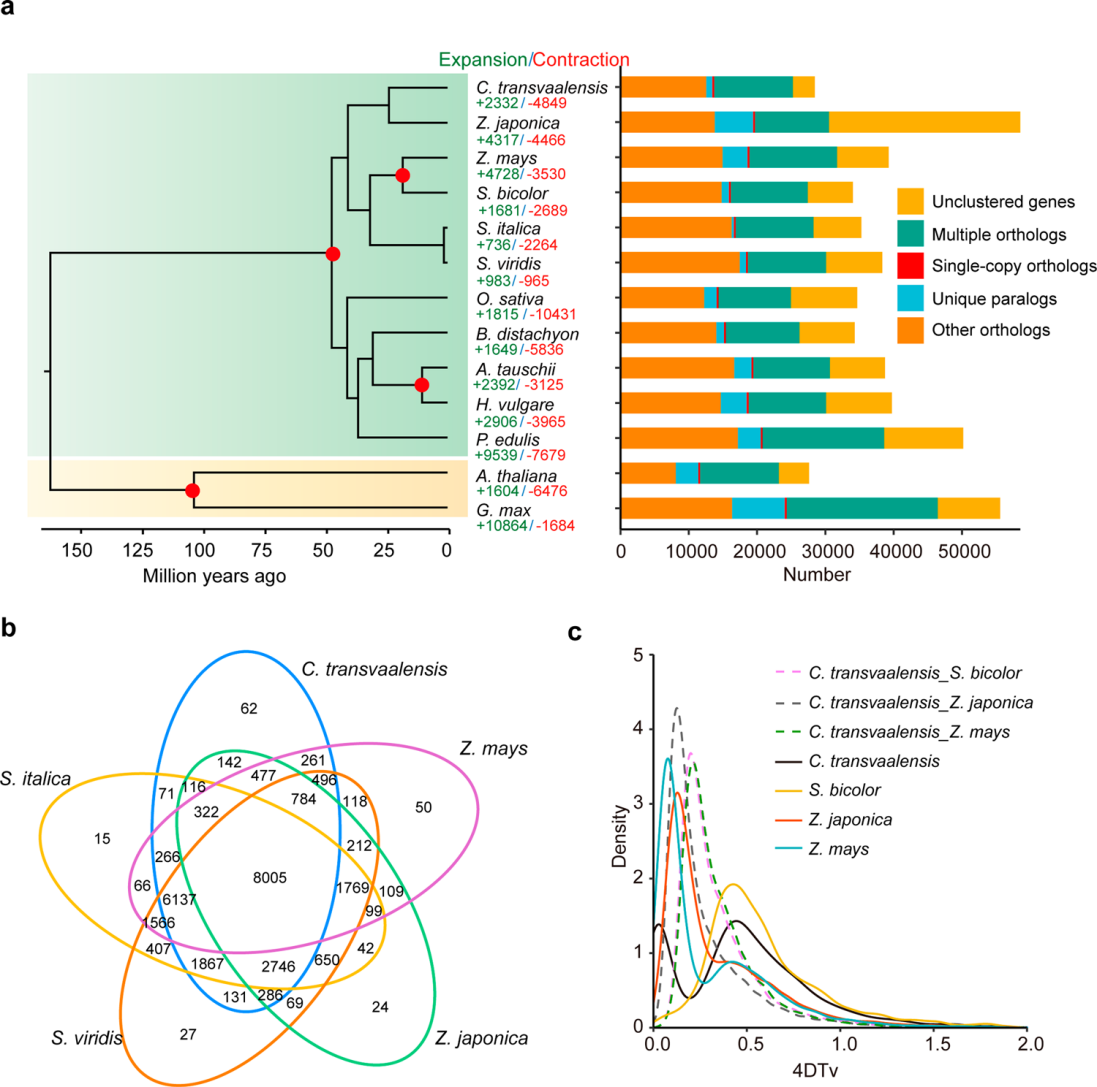
Gene family and phylogenetic tree analyses of *C. transvaalensis* and other representative plant genomes. **a** Gene family clustering in *C. transvaalensis* and twelve other plant genomes (right), gene family expansions and contractions among *C. transvaalensis* and twelve other species (middle) and a phylogenetic tree based on shared single-copy gene families (left). **b** Venn diagram of the number of shared gene families within *C. transvaalensis*, *S*. *italica*, *S*. *viridis*, *Z*. *japonica*, and *Z*. *mays*. **c** Distribution of fourfold degenerate sites of the third codons (4DTv) for syntenic genes within *C. transvaalensis*, *S*. *bicolor*, *Z*. *japonica*, and *Z*. *mays*

Gene family analysis was performed by comparing the African bermudagrass (*C. transvaalensis*) genome with those of twelve other representative plant species (*Aegilops tauschii*, *Arabidopsis thaliana*, *Brachypodium distachyon*, *Glycine max*, *Hordeum vulgare*, *Oryza sativa*, *Phyllostachys edulis*, *S. bicolor*, *S. italica*, *S. viridis*, *Z. japonica* and *Z. mays*). The results revealed that 25,260 genes in *C. transvaalensis* were clustered into 16,595 gene families, and 3184 genes were not clustered into any gene family. The clustered gene family number for *A*. *thaliana* was the lowest (12,775), while that of *S*. *viridis* (20,874) was the highest (Supplementary Table 8). Among the five species (*C. transvaalensis*, *Z*. *mays*, *Z*. *japonica, S*. *italica*, and *S*. *viridis*), the number of shared orthologous gene families was 8005, which was higher than that in *C. transvaalensis* and other species (Fig. 3b and Supplementary Fig. 7a, b). Then, 278 single-copy orthologous genes were utilized to construct the phylogenetic tree, with *A*. *thaliana* and *G*. *max* as outgroup species (Fig. 3a), and the tree indicated that the evolutionary relationship between *C. transvaalensis* and zoysiagrass was closer than that among other species. Based on the published fossilization time, the divergence time between *C. transvaalensis* and zoysiagrass was estimated to be ~23.6 million years ago (Mya).

Whole-genome duplication (WGD) events are an important driving force for plant evolution and adaptation to the environment. To explore the evolutionary history of the *C. transvaalensis* genome, fourfold degenerate sites of the third codons (4DTv) and synonymous substitutions per site (*Ks*) were calculated according to homologous and paralogous gene pairs. When the 4DTv peaks were 0.34–0.45, *C. transvaalensis*, maize, and sorghum experienced the shared WGD event. In addition, *C. transvaalensis* had a recent WGD event when the 4DTv was 0.03 (Fig. 3c), and the WGD event is shown in the circular diagram above (Fig. 2). During the evolution and speciation process, *C. transvaalensis* and zoysiagrass had a closer divergence time than *C. transvaalensis* and other species (*S*. *bicolor* and *Z. mays*), consistent with the findings from the phylogenetic tree. As expected, *Ks* distributions displayed trends similar to those of the 4DTv results (Supplementary Fig. 8). Positive selection analysis detected 28 genes (*Ka*/*Ks* > 1), most of which could be annotated to the Swiss-Prot database, such as genes involved in UV sensing, signal transduction, and oxidation-reduction (Supplementary Table 9).

### Genome synteny

**Fig. 4:**
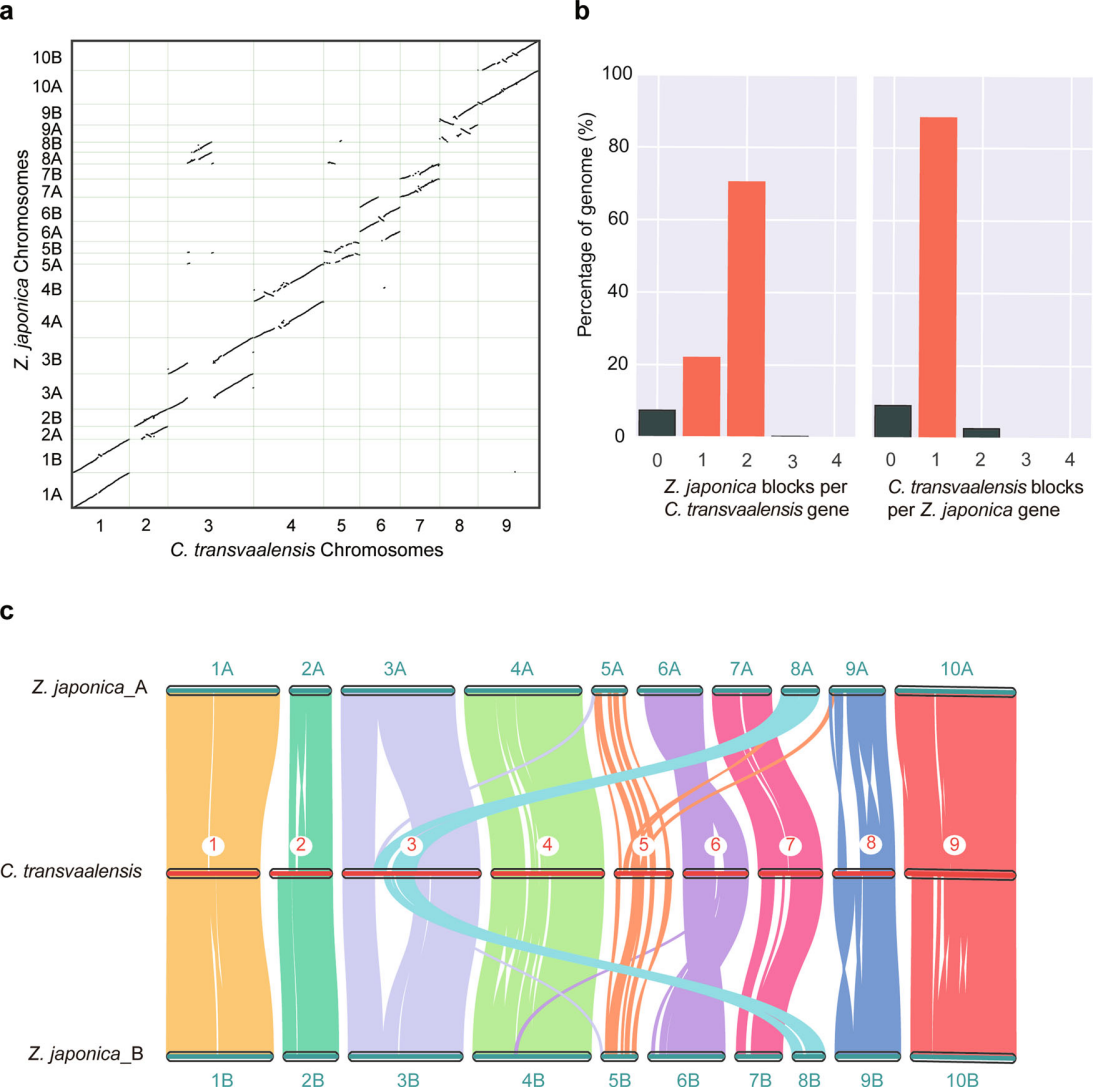
Genomic synteny analysis of *C. transvaalensis* and *Z. japonica*. **a** Dot plots for syntenic genes between *C. transvaalensis* and *Z*. *japonica*. **b** Synteny pattern in *C. transvaalensis* vs. *Z*. *japonica*. **c** Subgenome classification of *Z*. *japonica* (*Z*. *japonica* A and *Z*. *japonica* B) and synteny analysis

Based on the phylogenetic tree, genomic synteny analyses of *C. transvaalensis* and the other five species (*Z*. *mays*, *Z*. *japonica, S*. *bicolor*, *S*. *italica*, and *S*. *viridis*) were performed to explore their evolutionary relationships. Both *C. transvaalensis* and zoysiagrass were perennial warm-season grasses, and genomic dot plots showed good collinearity (Fig. 4a). The synteny pattern of these two species was 1-to-2 (Fig. 4b), and then, we defined two subgenomes (*Z*. *japonica* A and *Z*. *japonica* B) according to the synteny relationships displayed in Fig. 4c. Interestingly, compared with the most recent common ancestor (MRCA), *C. transvaalensis* and zoysiagrass might have experienced chromosome breakage-fusion events (*C. transvaalensis* Chr3 vs. zoysiagrass 8A and zoysiagrass Chr3 vs. zoysiagrass 8B), although most of the chromosomes had 1-to-1 collinearity. After speciation, intergenomic linear rearrangement events also caused some inversions (*C. transvaalensis* Chr6 vs. zoysiagrass 6A and zoysiagrass Chr6 vs. zoysiagrass 6B), which might have contributed to species specificity and environmental adaptation.

**Fig. 5:**
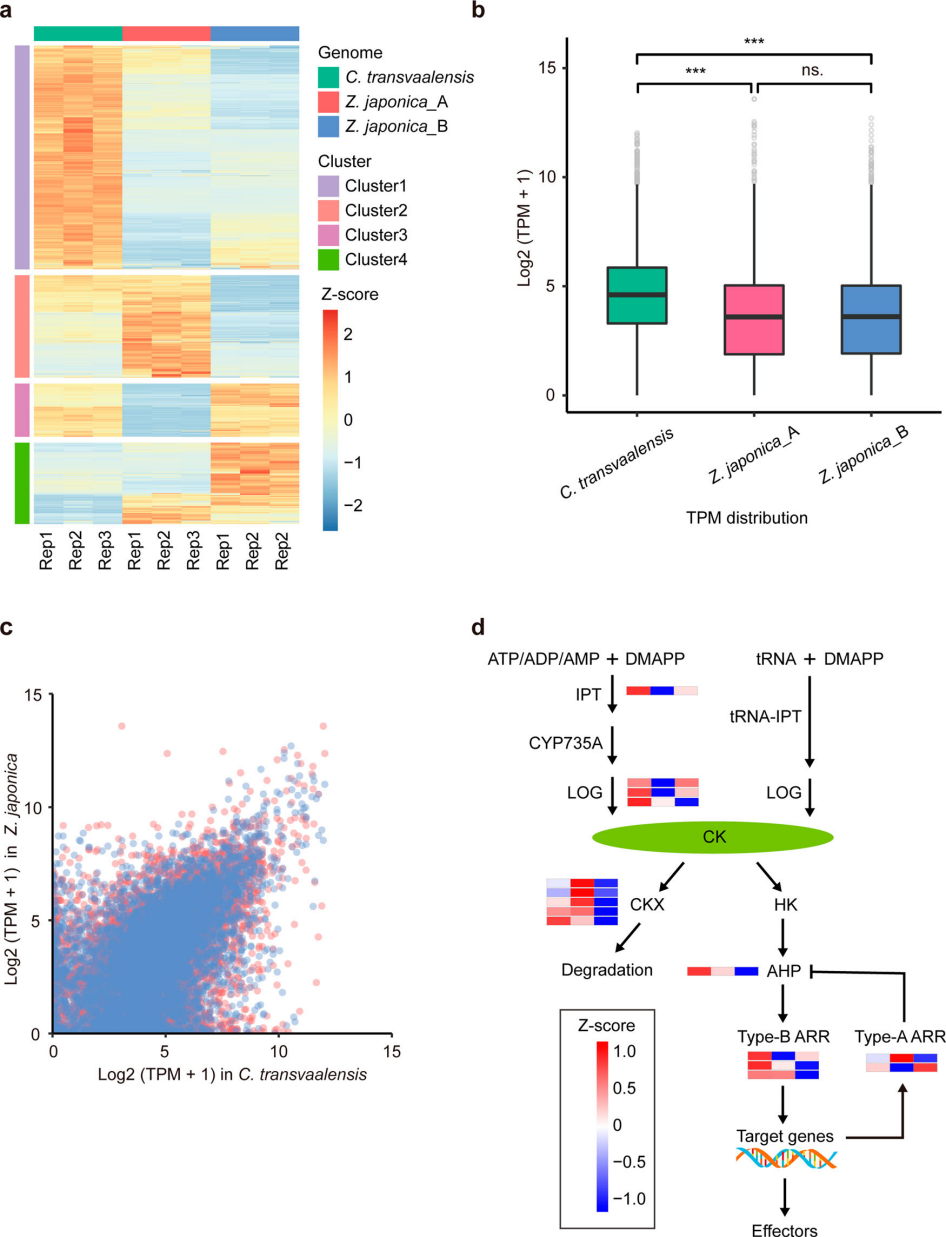
Gene expression pattern identification in *C. transvaalensis* and two subgenomes of zoysiagrass (*Z. japonica A* and *Z. japonica B*). **a** Gene expression heatmap of four clustered expression patterns. **b** Syntenic gene expression comparison within *C. transvaalensis*, *Z*. *japonica* A, and *Z*. *japonica* B. A nonparametric Wilcoxon rank-sum test was performed to evaluate significant differences for three pairs of expression levels. n.s. not significant; ***P < 0.01. **c** Syntenic gene dot plots for *C. transvaalensis* vs. *Z*. *japonica* A (red dots) and *C. transvaalensis* vs. *Z*. *japonica* B (blue dots). **d** Overview of cytokinin (CK) metabolism and signaling pathways showing the core genes enriched and their expression in *C. transvaalensis, Z*. *japonica* A, and *Z*. *japonica* B. Briefly, the first step of CK synthesis is that isopentenyladenine (iP) nucleotides are formed by IPT enzymes and then converted to the respective trans-zeatin (tZ) nucleotides by CYP735As. Activation of the inactive form of CKs is catalyzed by LOG enzymes, while the inactivation of all metabolites is catalyzed by CKX enzymes. Active CKs bind to HK receptors, thereby initiating phosphorelay signaling via HPTs to Type-B ARRs, which activate cytokinin response genes. Among these, Type-A ARRs are negative feedback regulators. DMAPP dimethylallyl diphosphate, IPT adenosine phosphate-isopentenyltransferase, tRNA-IPT tRNA-isopentenyltransferase, CYP735A cytochrome P450 monooxygenases, LOG cytokinin nucleoside 5′-monophosphate phosphoribohydrolase, CKX cytokinin oxidase/dehydrogenase, HK histidine kinase, AHP Arabidopsis histidine phosphotransferase, Type-B ARR Arabidopsis type-B response regulator, Type-A ARR Arabidopsis type-A response regulator

To examine the expression patterns, we identified 8816 syntenic genes in *C. transvaalensis* and two zoysiagrass subgenomes, and 6500 genes were retained after filtering low transcripts per million values according to leaf transcriptome sequencing data. Overall, the expression of *C. transvaalensis* was significantly higher than that in the two zoysiagrass subgenomes (Fig. 5b, c). Standardized gene expression (z-score) could be grouped into four clusters, each of which included a different number of genes (Cluster 1: 3158; Cluster 2: 1445; Cluster 3: 751; Cluster 4: 1146) and displayed a different expression pattern (Fig. 5a). From Cluster 1 to Cluster 4, the dominant species were *C. transvaalensis*, *Z*. *japonica* A, *Z*. *japonica* B, and *Z*. *japonica* B, respectively, which might suggest that although *C. transvaalensis* and zoysiagrass had a 1-to-2 synteny pattern, their expression levels were basically the same. The two subgenomes of zoysiagrass often exhibited complementary expression patterns, contributing to the balanced expression in the plants^37^. GO analyses were also performed for different clusters, and terms such as response to hormone, cytokinin (CK) metabolic process and catalytic activity were significantly enriched in clusters 1, 2, and 3, respectively (Supplementary Table 10). Here, we further compared the expression of several key genes involved in CK metabolism and signaling pathways^38,39^ among *C. transvaalensis*, *Z*. *japonica* A, and *Z*. *japonica* B, such as the adenosine phosphate-isopentenyltransferase (IPT), Arabidopsis histidine phosphotransferases (AHP), Arabidopsis type-B response regulators (Type-B ARR), and cytokinin oxidase/dehydrogenase (CKX) pathways. Notably, the overall CK pathway of *C. transvaalensis* was more active than that of *Z*. *japonica*, as demonstrated by the higher expression levels of positively regulated genes in the CK pathway (e.g., IPT, LONELY GUY (LOG), AHP, and Type-B ARR) and lower expression of negatively regulated genes (e.g., CKX, Type-A ARR) in *C. transvaalensis* (Fig. 5d). More interestingly, bermudagrasses are aggressive turfgrass species and can recover from foot traffic and other damage quickly, whereas zoysiagrasses are known for their slow growth and establishment rates^40^. Therefore, the growth rate difference between the two grasses might be partly attributable to the different regulation levels of CK metabolism and signaling pathways, considering the important role of CK in plant growth and development^38,39^. In general, CK-deficient plants develop stunted shoots, while accelerated plant growth rates are accompanied by high elevation of active CKs^41,42^.

For *C. transvaalensis* and the other four species (*Z*. *mays*, *S*. *bicolor*, *S*. *italica*, and *S*. *viridis*), we also performed synteny comparisons, and the results all showed high collinearity. The synteny pattern was 1-to-2 in *C. transvaalensis* vs. maize, which was likely because maize originated from allotetraploid ancestors^43^, and the patterns for *S. bicolor*, *S. italica*, and *S. viridis* were all 1-to-1 (Supplementary Fig. 9). Notably, zoysiagrass and sorghum had the same chromosome order in dot plots, indicating that there was no chromosomal rearrangement after speciation. The dot plots between zoysiagrass vs. *S*. *italica* and zoysiagrass vs. *S*. *viridis* were very similar because *S*. *italica* and *S*. *viridis* were closely related species with high genome consistency (Supplementary Fig. 10).

### Genome expansion and contraction

Genome expansion and contraction of *C. transvaalensis* were identified based on gene families. In total, 2332 expanded and 4849 contracted gene families were obtained, and the number of expanded genes was more than nine times the number of contracted genes (Fig. 3a). We also performed GO and KEGG analyses, and the GO results were mainly enriched in terms such as ADP-binding, ATPase activity, and oxidoreductase activity (Supplementary Table 11). KEGG pathways were mainly enriched in pathways such as MAPK signaling and glucose metabolism (amino sugar and nucleotide sugar metabolism, galactose metabolism, tryptophan metabolism, and glycolysis/gluconeogenesis) (Supplementary Table 12 and Supplementary Figs. 11 and 12). Overall, most of the expanded genes were involved in stress resistance and energy metabolism, which could help to increase the environmental adaptability of plants.

**Fig. 6:**
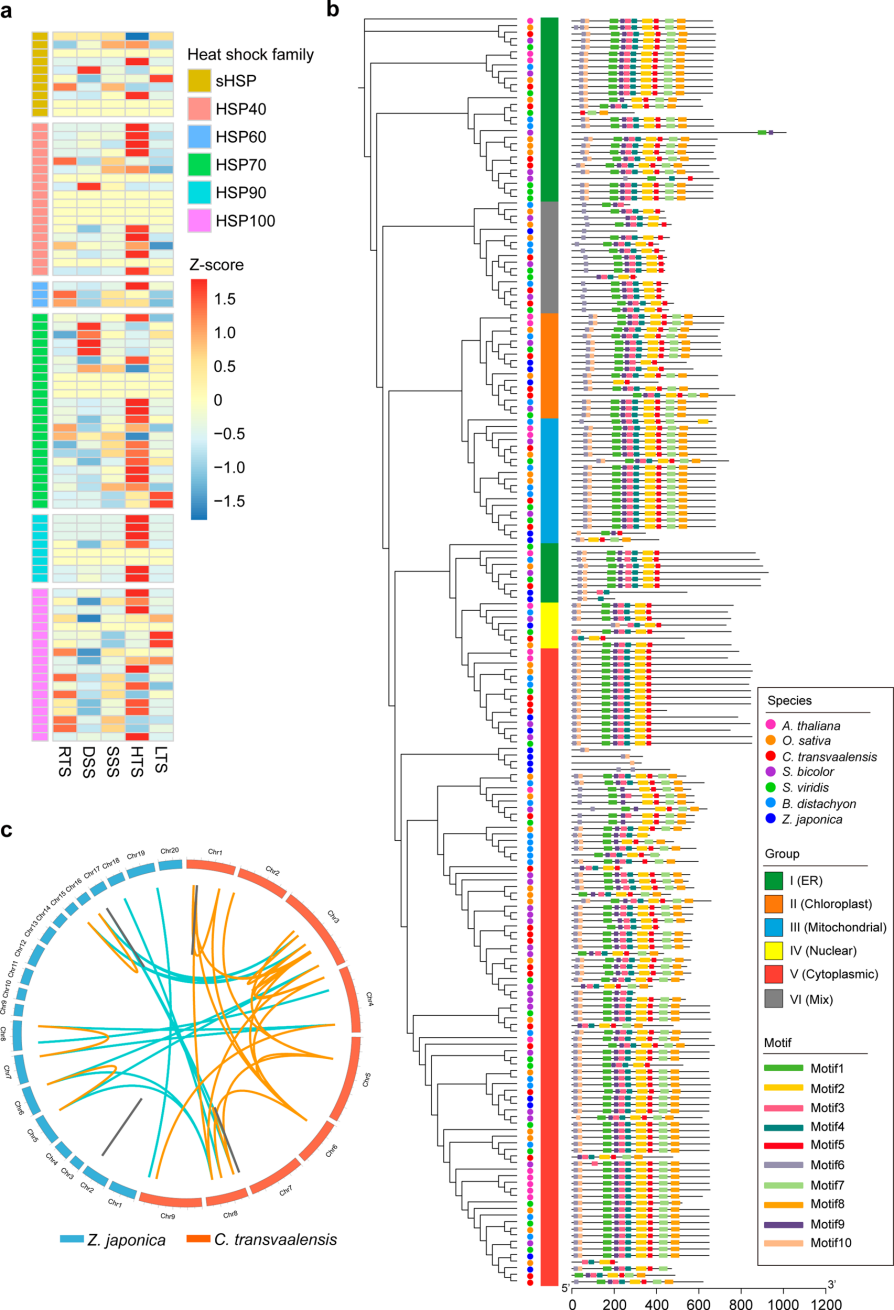
HSP family expression and expansion analysis. **a** Heatmap for expanded heat shock protein-encoding gene families. RTS, HTS, DSS, SSS, and LTS represent the shoot samples of *C. transvaalensis* under optimum temperature (25/30 °C, day/night, control), high temperature (45 °C for 6 h), drought stress (relative leaf water content of ~60%), salinity stress (200-mmol/L NaCl), and low temperature (4 °C for 6 h), respectively. **b** HSP70 family phylogenetic tree and motif patterns among seven species (*A. thaliana*, *B. distachyon*, *C. transvaalensis*, *O. sativa*, *S. bicolor*, *S. viridis*, *Z. japonica*). The unrooted tree was generated with IQ-TREE 2 software (http://www.iqtree.org/) using the full-length amino acid sequences of the 193 HSP70 proteins. The maximum-likelihood method was selected with 1000 bootstrap replicates. Subfamilies of HSP70s (I–VI) are highlighted with different colored vertical bars next to the gene names in the tree visualized with ITOL (https://itol.embl.de/). Motifs were identified by MEME (https://meme-suite.org/). The sizes of the HSP70 proteins and motifs can be estimated using the scale at the bottom. **c** Orthologous and paralogous *HSP70* gene pair links between *C. transvaalensis* and *Z. japonica*. Orange and gray links indicate intraspecies segmental and tandem duplications, and sky-blue links indicate interspecies orthologous gene pairs

Heat shock protein (HSP)-encoding gene families were among the significantly expanded gene families and contained 80 different *HSP* genes, and the majority of the *HSPs* exhibited a different expression pattern in response to abiotic stresses (Fig. 6a and Supplementary Table 13). Subsequently, the HSP70 family was chosen from the six HSP families as an example for phylogenetic and protein motif analysis, and 193 *HSP70* genes were identified among seven species, namely, C. transvaalensis (32), *A. thaliana* (18), *B. distachyon* (30), *O*. *sativa* (32), *S. bicolor* (33), *S. viridis* (29), and *Z. japonica* (19), with conserved HSP70 domains. According to the predicted protein subcellular locations^44^, the phylogenetic tree classified the HSP70s into six groups (I–VI) (Fig. 6b and Supplementary Table 14). The tree also revealed orthologous and paralogous relationships among the HSP70 members of the seven species. Notably, in addition to the 18 orthologous *HSP70* gene pairs between *C. transvaalensis* and zoysiagrass, there were 24 and 7 paralogous pairs in *C. transvaalensis* (4 tandem and 20 segmental duplicated pairs) and zoysiagrass (2 tandem and 5 segmental duplicated pairs), respectively (Fig. 6c and Supplementary Tables 15, 16). These results supported not only the relatively close relationship between the two Chloridoideae species, namely, *C. transvaalensis* and zoysiagrass, but also the respective occurrence of species-specific *HSP70* gene duplication events in both species, particularly the speculated genome-wide duplication event in *C. transvaalensis* after the divergence of the two species (Fig. 3c). Similar expansion mechanisms of the HSP70 family, such as tandem and segmental gene duplication, were also found in other plant species^45,46^, which is among the major forces eventually driving the evolution of plant genomes^47,48^.

In addition, several fatty acid biosynthetic process-encoding gene families were also expanded, such as the 3-ketoacyl-CoA synthase (KCS), palmitoyl-acyl-carrier protein thioesterase, and 3-oxoacyl-[acyl-carrier-protein] synthase (Supplementary Table 17) families. A total of 13 out of the 19 *KCS* genes were upregulated by either drought or heat stress, but nearly none of them were upregulated under low temperature (Supplementary Fig. 13). For contracted gene families, KEGG pathways were significantly enriched in phenylpropanoid biosynthesis (Supplementary Table 18), which could affect lignin content via both syringyl and guaiacyl monomers according to the pathway map (Supplementary Fig. 14)^49^. A previous study found that the lignin content in C. transvaalensis was less than that in zoysiagrass; therefore, C. transvaalensis could potentially regulate the lignin content to provide more readily digestible forage for animals^50^.

## Discussion

Notably, the estimated genome sizes of C. transvaalensis determined by k-mer analysis and flow cytometry in this study were 454.4 and 444.3 Mb (Supplementary Figs. 1 and 2), respectively, which were ~8–17% smaller than the previously reported estimation (~495–540 Mb)^8,28,51^. We also estimated the genome size of common bermudagrass (2*n* = 4*x* = 36) using flow cytometry analysis. As expected, its genome (~905.3 Mb, Supplementary Fig. 2) was nearly double the size of the diploid African bermudagrass used in the study^28^. We postulated that the smaller genome size might be due to internal standard differences, experimental errors, or other differences among studies.

Before this study, zoysiagrass (*Z. japonica*) was the first reported turfgrass species with a relatively high-quality draft genome (e.g., contig N50 value = 2.4 Mb, covering 85.6% of the estimated genome), but its coverage at the chromosome scale was poor (70.2%) due to the limitations of short-read Illumina sequencing, the scaffolding method used (genetic linkage map) and its genome properties (e.g., allotetraploid, high proportion of heterozygous alleles)^25^. Here, we report the first high-quality chromosome-scale genome assembly (contig N50 value = 2.6 Mb, covering 93.2% and 84.3% of the estimated genome at the contig and scaffold levels, respectively, and annotated gene BUSCO value = 96.73%) of a turfgrass species (Supplementary Fig. 15), C. transvaalensis, using a combination of Nanopore sequencing, BioNano optical mapping, and Hi-C scaffolding. It is also the first reference genome of the genus *Cynodon*; this genome is superior to the recently published genomes of several other Chloridoideae subfamily species, such as goosegrass (N50 value = 32.5 kb, BUSCO value = 95%)^24^, finger millet (N50 value = 23.7 kb)^23^, tef (N50 value = 85 kb)^27^, *Eragrostis curvula* grass (N50 value = 380 kb, BUSCO value = 96%)^26^, and *Oropetium thomaeum* (V1 assembly, N50 value = 2.4 Mb, BUSCO value = 72%)^32,33^. As the 3rd chromosome-scale assembly among the Chloridoideae grasses, the pseudomolecules in the C. transvaalensis reference genome would facilitate further comparative genomic and quantitative genetic studies, such as more accurate identification of syntenic orthologs in other grasses and more detailed comparisons of chromosome evolution in grasses, particularly Chloridoideae species^33^.

Genomic collinearity is very common in grass families^52^. In this study, we compared the genome of C. transvaalensis with that of Z. japonica, and as expected, C. transvaalensis showed a high genomic syntenic relationship with Z. japonica due to their close phylogenetic relationship within the Chloridoideae subfamily (Figs. 3 and 4). Similarly, another Cynodon species, C. dactylon, was reported to have a high degree of chromosome-level collinearity with two Chloridoideae grasses, including Z. japonica^53^. According to the phylogenetic analysis, C. transvaalensis and Z. japonica diverged from the MRCA ∼23.6 Mya, and they shared an MRCA with the Panicoideae subfamily species (*S. bicolor*, *S. italica*, *S. viridis*, and *Z. mays*) belonging to the “PACMAD” clade ∼40.6 Mya, whereas ∼47.3 Mya, they shared an MRCS with Bambusoideae (*P. edulis*), Oryzoideae (*O. sativa*), and Pooideae subfamily species (*A. tauschii*, *B. distachyon, H. vulgare*) belonging to the “BOP” clade (Bambusoideae, Oryzoideae, and Pooideae), which is consistent with previous studies^17,53,54^.

As a C_4_ perennial grass species, unlike its ubiquitous counterpart C. dactylon, the native distribution of C. transvaalensis is nearly completely confined to South Africa, which makes it of particular interest for investigation of its evolution and adaptation to the environment, such as the tropical and/or subtropical climate, typically with dry summers, since its origination^30^. Gene family expansion and contraction due to selection pressure play important roles in the adaptation and evolution of species^55–57^. All six families of HSPs were expanded (Fig. 6), including the small HSPs, HSP40/DnaJ, HSP60/GroEl, HSP70/DnaK, HSP90/HtpG, and HSP100/Clp, which are generally grouped based on their approximate molecular weight^58,59^. HSPs usually protect cells against various abiotic stresses by functioning mainly as molecular chaperones to control the proper folding and conformation of proteins; in particular, numerous studies have reported their important roles in plant heat stress tolerance^60–63^. For instance, HSP101 is a key component for thermotolerance acquisition in plants^64^. The expansion of the HSP families of *C. transvaalensis* indicated possible evolution and adaptation to warm/high temperatures. Moreover, the transcriptome data here showed that the majority of the expanded HSPs were induced under high temperature, and some of them were induced by drought, salinity, or even low temperature (Fig. 6a), which further indicated their major roles in the response to heat stress and possibly some roles in the responses to other abiotic stresses^65^. Several genomic studies have also reported the expansion features of some HSP families, which are thought to be related to differences in environmental adaptation^57,66,67^.

In addition to the HSP families, several families of genes involved in fatty acid biosynthetic processes were also expanded, including the KCS family (Supplementary Table 17). KCS genes are known to contribute to very-long-chain fatty acid (VLCFA) synthesis and to play an important role in wax biosynthesis^68,69^; thus, they are involved in plant drought tolerance^70–72^. More gene copies of KCS might help *C. transvaalensis* reduce water loss and better confront dry climates by altering VLCFA biosynthesis. Some bermudagrass species are able to form a layer of wax on the leaf surface to adapt to osmotic stress^73^. The leaf wax layer can also protect plants from strong UV irradiation^74,75^, such as the condition faced by *C. transvaalensis* during the dry and hot summer in Africa. In addition, UVR8, a photoreceptor for ultraviolet B that triggers a signaling pathway for ultraviolet protection^76^, was among the 28 positively selected genes (Supplementary Table 9), indicating the adaptation of *C. transvaalensis* to a high-ultraviolet environment. Similar gene selection and UV adaptation mechanisms were also reported in other plant species, such as *Juglans sigillata*^77^ and maca (*Lepidium meyenii*)^78^ growing at high altitudes.

In summary, here, we report the first chromosome-level reference genome in the genus *Cynodon*, a high-quality genome assembly of *C. transvaalensis* determined using several types of sequencing data and assembly methods. Evolutionary analysis and transcriptome comparison showed a preliminary genomic basis for its adaptation to a warm climate with a dry summer environment. The genomic resources generated in this study will not only facilitate further evolutionary studies of the Chloridoideae subfamily but also provide valuable resources for functional genomic research and genetic breeding of new bermudagrass cultivars to be used as turfgrass in the future.

## Materials and methods

### DNA isolation and sequencing

Leaf samples from single stolon-propagated C. transvaalensis plants grown in the greenhouse of China Agricultural University, Beijing, China, were collected and frozen in liquid nitrogen. Genomic DNA was extracted by the QIAGEN^®^ Genomic DNA Extraction Kit (Qiagen, Hilden, Germany), and the size was selected using the BluePippin system (Sage Science, USA). The selected and purified DNA was then prepared for sequencing following the protocol provided with the genomic sequencing kit SQK-LSK109 (Oxford Nanopore Technologies, Oxford, UK). Single-molecule real-time sequencing was conducted on a Nanopore GridION X5 platform by NextOmics Biosciences (Wuhan, China). Before assembly, reads of low quality, reads shorter than 2000 nt, and reads with adapters were filtered out. Then, 2000 randomly selected reads were subjected to BLAST+ 2.9.0 searches against the NT database, and no obvious external contamination was found. For Illumina sequencing, a separate paired-end library with an insert size of 350 bp was constructed and sequenced by NextOmics Biosciences in accordance with the manufacturer’s protocol using the Illumina NovaSeq platform (Illumina, Inc., San Diego, USA) with 150-bp paired-end reads. A total of 19.44 Gb of Illumina raw sequencing data was generated for the survey of C. transvaalensis, and the data were also used for correction and accuracy evaluation of the genome of C. transvaalensis.

### Genome survey of African bermudagrass and common bermudagrass

The genome size of C. transvaalensis was estimated using both flow cytometry (EPICS XL, Beckman Coulter, Inc., CA, USA), with sorghum (*Sorghum bicolor*) “BTx623,” maize (*Zea mays*) “B73,” and rice (*Oryza sativa*) “Nipponbare” as the internal standards^28,79^, and the *k*-mer method^80^ using Illumina genomic DNA sequencing data. Quality-filtered reads were subjected to 17-mer frequency distribution analysis using the Jellyfish 2.0 program (www.genome.umd.edu/jellyfish.html). The genome size (*G*) of C. transvaalensis was estimated using the following formula: *G* = *K*_num_/*K*_depth_ = *b*_num_/*b*_depth_, where *K*_num_ is the number of *k*-mers, *K*_depth_ is the expected depth of *k*-mers, *b*_num_ is the base number, and *b*_depth_ is the expected base depth. The count distribution of the 17-mer followed a Poisson distribution, with two peaks occurring at depths of 17 and 36 (Supplementary Fig. 1).

### Genome assembly, polishing, and quality evaluation

Several genome assembly software programs were applied to assemble the genome of C. transvaalensis preliminarily, including wtdbg2 v2.5 (-g 450m -x ont)^81^, smartdenovo v1.0 (-k 21)^82^, and Nextdenovo (read_cutoff = 1k; seed_cutoff = 25k) (https://github.com/Nextomics/NextDenovo). The quality-controlled Nanopore reads were corrected with Nextdenovo and assembled with Smartdenovo for further genome assembly with the same parameters as those described above. Illumina short reads were mapped to the preliminary genome assembly using minimap2 (v0.7.12-r1039, default parameters)^83^, and the reads were then used to polish the assembly four times with NextPolish (v1.0.5, -max_depth 100)^84^. Finally, BWA MEM (http://bio-bwa.sourceforge.net/) and BUSCO v3.0.1^36^ were used to assess the quality and completeness of the assembly.

### BioNano library construction, sequencing, and scaffolding

High-molecular-mass genomic DNA was isolated as previously described^32,85,86^. Briefly, ~3 g of fresh young leaves of C. transvaalensis was ground to a fine powder in liquid nitrogen and transferred to a 50-ml Falcon tube. The sample was filtered through one layer of Miracloth and two layers of cheesecloth. Nuclei were purified with Percoll gradients and washed extensively before being embedded in 60 µl of low-melting agarose. The DNA plugs were then treated overnight with 500 µl of lysis buffer containing proteinase K, detergent, and β-mercaptoethanol, followed by digestion in 100 µl of RNase A for 1 h. DNA was extracted from the samples, purified, and quantified with a Qubit 3.0 fluorometer. Finally, ~600 ng of genomic DNA was subjected to labeling with the SaphyrPrep Reagent Kit (BioNano Genomics, San Diego, USA). The labeled library was loaded onto a Saphyr Chip and imaged on a Saphyr imaging instrument (BioNano Genomics). Molecules collected from BioNano chips were filtered using a molecule length cutoff of <150 kb and/or a molecule minSites cutoff of <9, and 52.6 Gb of clean data was aligned to the Nanopore genome assembly to generate a molecular quality report, yielding a mapping rate of 45%. To further obtain a longer scaffold, the de novo assembly of Nanopore reads was mapped to the BioNano single-molecule genomic map as previously described^77,87^ using the Bionano Access 1.1.2 and Bionano Solve 3.2 hybrid-scaffolding pipeline (hybrid-scaffolding parameters: nonhaplotype without extension and splitting).

### Hi-C data analysis and pseudochromosome construction

The Hi-C library was prepared according to Belton et al.^88^ and Shi et al.^89^ with a modification. In brief, freshly harvested young leaves of C. transvaalensis plants were cut into ~2-cm pieces and fixed in nucleus isolation buffer with 2% formaldehyde after vacuum infiltration for 15 min. The chromatin crosslinking reaction was stopped by adding glycine, and the fixed tissue was then collected, frozen in liquid nitrogen, and ground to powder for nucleus isolation. DNA was extracted from the isolated nuclei, purified, and digested with 100 units of HindIII, and the crosslinked fragments were labeled at the ends with biotin-14-dCTP. The ligated DNA was sheared into 300- to 600-bp fragments and then blunt-end repaired and A-tailed, followed by purification through biotin-streptavidin-mediated pull-down. The purified DNA fragments were used for Hi-C library construction. The libraries were quantified and sequenced on the Illumina NovaSeq platform using the PE 150 layout, which yielded ~93.6 Gb of data with 624 million paired-end reads. Then, quality control of Hi-C raw data was performed using HiC-Pro (v2.8.0) as described by Burton et al.^90^. First, low-quality sequences (quality scores <20), sequences with adapters and sequences shorter than 30 bp were filtered out using fastp v0.12.6^91^, and then, the 265 million clean paired-end reads were mapped to the assembled draft sequence using Bowtie2 (v2.3.2) to obtain the unique mapped paired-end reads^92^. As a result, 87 million uniquely mapped paired-end reads were generated, of which 70.34% (61.5 million) were valid interaction pairs. In combination with the valid Hi-C data, we subsequently used the LACHESIS (ligating adjacent chromatin enables scaffolding in situ) de novo assembly pipeline^93^ to generate chromosome-level scaffolds of the C. transvaalensis genome.

### Genome repeat element identification and gene function annotation

In order to annotate the repeat sequences in C. transvaalensis, based on the principle of repeat sequence-specific structure and de novo prediction, we first used the software LTR_FINDER^94^, MITE-Hunter^95^, and RepeatModeler (www.repeatmasker.org/RepeatModeler/) to build a repeat sequence database and then combined it with the Repbase database^96^ to form the final repeat sequence database. Finally, repeat sequence prediction of the C. transvaalensis genome was performed by using the software RepeatMasker (www.repeatmasker.org) with the final repetitive sequence data as an index file.

For prediction and annotation of the protein-coding genes, the automated eukaryotic gene structure annotation tool EVidenceModeler (EVM)^97^ was used by combining three approaches: de novo gene prediction with Augustus^98^, de novo assembly of transcripts from RNA-seq data with Transdecoder (https://github.com/TransDecoder/TransDecoder/releases), and prediction of homologous proteins with GeMoMa^99^. Then, TransposonPSI (http://transposonpsi.sourceforge.net) was used to identify potential transposon ORFs within the gene set with PSI-Blast, and the genes were filtered out from the final gene set. Following annotation, singleton and duplicated genes were identified with MCscan^100^, and WGD events were visualized with Circos (http://circos.ca/)^101^. The longest protein sequences for each gene were subjected to BLAST analysis against the Swiss-Prot, Nr, KEGG, and KOG/COG databases to identify homologous proteins using Blastp with default parameters, except *E* value ≤ 1e − 5. The functional domains and possible GO terms in the protein sequences were identified with InterProScan (http://www.ebi.ac.uk/interpro/) with -goterms -t p -f GFF3 -pa -cpu 100. BUSCO evaluation was used to further verify the gene annotation results by searching against the embryophyta_odb10 database.

OrthoMCL^102^ (http://orthomcl.org/orthomcl/, v2.09) with the parameters -abc -I 1.5 was used to cluster the gene families for the thirteen species (*A. tauschii*, *A. thaliana*, *B. distachyon*, *C. transvaalensis, G. max*, *H. vulgare*, *O. sativa*, *P. edulis*, *S. bicolor*, *S. italica*, *S. viridis*, *Z. japonica*, and *Z. mays*) herein. The protein sequences of the genes from 278 single-copy gene families for the 13 species described above were aligned using MAFFT^103^ and then converted into a coding sequence (CDS) alignment. The poorly aligned CDS regions were filtered out with Gblocks^104^, and the RaxML method^105^ was used to construct a phylogenetic tree with the GTRGAMMA model (Bootstrap = 100). Among the 13 species, *A. thaliana* and *G. max* were used as the outgroup species.

Based on the gene family alignment data, the divergence times of C. transvaalensis from the other plants were estimated using MCMCTREE in the PAML software package^106^, and the times were further calibrated using the predicted divergence times of *Z. mays* and *S. bicolor* (11.73–33.32 Mya), *A. thaliana* and *G. max* (97.08–109.04 Mya), and *A. tauschii* and *H. vulgare* (7.75–13.54 Mya) based on available Timetree (http://timetree.org) fossil records.

The 278 common single-copy gene families in all 13 species were used to calculate the expanded and contracted gene families for each lineage using CAFÉ with the default parameters^107^. The orthologous genes among all 13 species were identified using Blastp with an *E* value ≤ 1e − 5, and then, genes under positive selection in the C. transvaalensis lineage were further identified based on the orthologous gene information using Codeml implemented in the PAML software package^106^. WGD was displayed using 4DTv (fourfold synonymous third-codon transversion). MCscan^100^ was used to identify syntenic regions and generate a synteny plot between C. transvaalensis and *Z. japonica*. The 4DTv value was calculated to predict WGD events, and the ratio of nonsynonymous (*Ka*) to synonymous (*Ks*) nucleotide substitution rates was calculated using Kaks_calculator^108^.

### RNA-seq analysis and KEGG pathway enrichment

Total RNA was extracted from leaves of C. transvaalensis under optimum growth chamber conditions [30/25 °C day/light, photosynthetically active radiation 800 µmol/m^2^/s], drought stress (after water withholding for 5 days with a relative leaf water content of ~60%), heat stress (45 °C for 6 h), cold stress (4 °C for 6 h), and salinity stress (200-mM NaCl for 24 h, soil salinity level was increased by 50 mM daily) with three biological replicates for Illumina RNA-seq. A total amount of 1 μg of RNA per sample was used for RNA-seq library preparation using TruSeq RNA Sample Prep Kits (Illumina) according to the manufacturer’s instructions. The 15 libraries were sequenced on an Illumina NovaSeq platform, and 150-bp paired-end reads were generated. Raw Illumina RNA-seq data/reads were trimmed for quality using fastp (https://github.com/OpenGene/fastp), and the quality of the resulting trimmed reads was further evaluated using FastQC (http://www.bioinformatics.babraham.ac.uk/projects/fastqc). For PacBio full-length transcriptome sequencing, equal amounts of the total RNA extracted with TRIzol reagent (Tiangen Biotech, Beijing, China) from each sample of leaves, stems, and roots were pooled together and used for library preparation. The Iso-Seq library was prepared using the Isoform Sequencing protocol with the SMARTer PCR cDNA Synthesis Kit (Takara, Dalian, China) and the BluePippin Size Selection System protocol (Sage Science, Beverly, USA) with some modifications by NextOmic Biosciences (Wuhan, China). PacBio sequencing was then performed on the PacBio Sequel platform according to the manufacturer’s protocol. HISAT2 v2.1.0^109^ was applied to map the quality-filtered data to the genome of C. transvaalensis assembled here, and StringTie v1.3.6^110^ was used to calculate gene expression. GO and KEGG pathway enrichment analysis was performed by clusterProfiler^111^.

## Supplementary information

Supplemental Figures and Tables of C. transvaalensis-2021-02-01

## Data Availability

The Nanopore long reads and Illumina short reads were uploaded to the China National Center for Bioinformation GSA (Genome Sequence Archive) database under BioProject PRJCA003581, and the submission ID is subPRO005221.
