# Supplementary material for: The genome of the warm-season turfgrass African bermudagrass (Cynodon transvaalensis)
Source: Hortic Res. 2021 May 1;8:93. doi: 10.1038/s41438-021-00519-w (PMC8087826; doi:10.1038/s41438-021-00519-w)
Supplement: Supplementary file 1 — Supplemental Figures and Tables of C. transvaalensis-2021-02-01 [file 41438_2021_519_MOESM1_ESM.docx]

**Supplementary Table 1. Summary of sequencing data used for genome assembly.**

| **Platform** | **Illumina** | **Nanopore** | **Bionano** | **Hi-C** |
| --- | --- | --- | --- | --- |
| Reads number (M) | ~126.47 | ~3.95 | ~0.18 | ~530.08 |
| Data volume (Gb) | ~18.80 | ~80.13 | ~52.60 | ~77.30 |
| Reads length (bp) | PE 150 | - | - | PE 150 |
| N50 value | - | ~28 kb | ~3.39 Mb | - |
| Coverage depth (×) | ~41.4 | ~176.3 | ~115.8 | ~170.1 |
| PE, paired-end. |  |  |  |  |

**Supplementary Table 2. Bionao molecule quality report (MQR).**

| **Sample** | ***C. transvaalensis*** |
| --- | --- |
| Total DNA (>= 20 kb) | 98.99 Gb |
| N50 (>= 20 kb) | 0.16 Mb |
| Total DNA (>=150 kb) | 52.60 Gb |
| N50 (>=150 kb) | 0.32 Mb |
| Total DNA (>= 150 kb & minSites >= 9) | 39.98 Gb |
| N50 (>=150 kb & minSites >= 9) | 3.39 Mb |
| Enzyme | DLE-1 |
| Enzyme recognition sequence | CTTAAG |
| Label color | Green_01 |
| Average label density (>= 150 kb) | 9.22/100 kb |
| Map rate (>= 150 kb) | 45.0% Percentage of molecules aligned to reference |
| Effective coverage | 44.19× Effective molecule coverage |
| Positive label variance (PLV) | 10.0% Percentage of labels absent in reference |
| Negative label variance (NLV) | 13.5% Percentage of reference labels absent in molecules |
| SiteSD | 0.09 Constant term in sizing error relative to reference |
| ScalingSD | 0.0000 Linear term in sizing error relative to reference |
| RelativeSD | 0.0378 Quadratic term in sizing error relative to reference |
| SMin | 0.09kb Minimum expected sizing error relative to reference |
| Base pairs per pixel | 463.75 Base pairs per pixel |

**Supplementary Table 3. Assembled genome BUSCO results.**

| **Type** | **Number** | **Percent (%)** |
| --- | --- | --- |
| Complete BUSCOs (C) | 1,334 | 97.0 |
| Complete and single-copy BUSCOs (S) | 1,187 | 86.3 |
| Complete and duplicated BUSCOs (D) | 147 | 10.7 |
| Fragmented BUSCOs (F) | 13 | 0.9 |
| Missing BUSCOs (M) | 28 | 2.1 |
| Total BUSCO groups searched | 1,375 | 100 |

**Supplementary Table 4. Repeat sequences identification and classification in genome.**

| **Class** | **Order** | **Type** | **Number** | **Length (bp)** | **Percent in genome (%)** |
| --- | --- | --- | --- | --- | --- |
| Class I |  |  | 165,527 | 95,922,801 | 25.04 |
|  | LTR |  | 142,297 | 84,799,001 | 22.14 |
|  |  | Unknown | 51,185 | 18,847,243 | 4.92 |
|  |  | Gypsy | 70,066 | 56,875,967 | 14.85 |
|  |  | Copia | 19,310 | 8,241,041 | 2.15 |
|  |  | Caulimovirus | 743 | 541,315 | 0.14 |
|  |  | Other | 993 | 293,435 | 0.08 |
|  | LINE |  | 23,007 | 11,104,041 | 2.9 |
|  |  | L1 | 20,842 | 10,726,190 | 2.8 |
|  |  | Other | 2,165 | 377,851 | 0.1 |
|  | SINE |  | 223 | 19,759 | 0.01 |
|  |  | Other | 223 | 19,759 | 0.01 |
| Class II |  |  | 147,437 | 32,217,314 | 8.41 |
|  | DNA |  | 128,309 | 28,693,508 | 7.49 |
|  |  | TcMar-Stowaway | 27,732 | 4,763,060 | 1.24 |
|  |  | CMC-EnSpm | 21,342 | 6,557,802 | 1.71 |
|  |  | MULE-MuDR | 26,684 | 5,710,351 | 1.49 |
|  |  | PIF-Harbinger | 30,641 | 6,744,017 | 1.76 |
|  |  | hAT-Ac | 8,028 | 1,986,589 | 0.52 |
|  |  | Unknown | 4,656 | 821,618 | 0.21 |
|  |  | hAT-Tag1 | 2,410 | 390,748 | 0.1 |
|  |  | hAT-Tip100 | 4,091 | 850,792 | 0.22 |
|  |  | MuLE-MuDR | 1,051 | 575,831 | 0.15 |
|  |  | Other | 1,674 | 292,700 | 0.08 |
|  | MITE |  | 8,344 | 1,801,569 | 0.47 |
|  |  | Unknown | 8,344 | 1,801,569 | 0.47 |
|  | RC |  | 10,784 | 1,722,237 | 0.45 |
|  |  | Helitron | 10,748 | 1,720,575 | 0.45 |
|  |  | Other | 36 | 1,662 | 0 |
|  | Total TEs |  | 312,964 | 128,140,115 | 33.45 |
|  | Unknown |  | 74,270 | 16,862,618 | 4.4 |
|  | Other |  | 3,761 | 882,562 | 0.23 |
|  | Simple repeats |  | 670 | 84,298 | 0.02 |
|  | Low complexity |  | 21 | 2,750 | 0 |
|  | Total repeats |  | 391,686 | 145,972,343 | 38.11 |

**Supplementary Table 5. Statistical information of SSR in assembled genome.**

| **Unit Size (repeat number)** | **Number** |
| --- | --- |
| 1 (>=10) | 82,725 |
| 2 (>=6) | 22,497 |
| 3 (>=5) | 11,526 |
| 4 (>=5) | 735 |
| 5 (>=5) | 207 |
| 6 (>=5) | 78 |

**Supplementary Table 6. Summary of sequencing data used for gene prediction.**

| **Platform** | **Pacbio** | **Illumina** |
| --- | --- | --- |
| Reads number (M) | ~15.26 | ~1,119.61 |
| Data volume (Gb) | ~26.26 | ~167.16 |
| Mean reads length (bp) | ~1,721 | PE 150 |
| N50 value (bp) | 1,971 | - |
| Max read length (bp) | 243,078 | - |
| PE, paired-end. |  |  |

**Supplementary Table 7. ncRNA (non-coding RNA) annotations in assembled genome.**

| **Type** | **Copy Number** | **Average Length (bp)** | **Total Length (bp)** | **Percent of Genome (%)** |
| --- | --- | --- | --- | --- |
| rRNA | 62 | 1183.02 | 73,347 | 0.0191 |
| tRNA | 760 | 74.99 | 56,995 | 0.0149 |
| miRNA | 803 | 122.89 | 98,677 | 0.0258 |
| regulatory | 15 | 56.67 | 850 | 0.0002 |

**Supplementary Table 8. Gene family identification and classification in multiple genomes.**

| **Species** | **Genes Number** | **Genes In Families** | **Unclustered Genes** | **Family Number** | **Unique Families Number** | **Average Genes Number Per Family** |
| --- | --- | --- | --- | --- | --- | --- |
| *A. tauschii* | 38,733 | 30,663 | 8,070 | 18,495 | 734 | 1.66 |
| *A. thaliana* | 27,615 | 23,196 | 4,419 | 12,775 | 907 | 1.82 |
| *B. distachyon* | 34,303 | 26,216 | 8,087 | 17,681 | 421 | 1.48 |
| *G. max* | 55,589 | 46,454 | 9,135 | 14,620 | 2,318 | 3.18 |
| *H. vulgare* | 39,734 | 30,108 | 9,626 | 17,769 | 1,173 | 1.69 |
| *O. sativa* | 34,664 | 24,954 | 9,710 | 17,394 | 710 | 1.43 |
| *P. edulis* | 50,180 | 38,624 | 11,556 | 17,715 | 1,267 | 2.18 |
| *S. bicolor* | 34,027 | 27,431 | 6,596 | 18,660 | 409 | 1.47 |
| *S. italica* | 35,270 | 28,277 | 6,993 | 19,729 | 106 | 1.43 |
| *S. viridis* | 38,334 | 30,119 | 8,215 | 20,874 | 303 | 1.44 |
| *Z. japonica* | 58,532 | 30,531 | 28,001 | 17,596 | 1,600 | 1.74 |
| *Z. mays* | 39,272 | 31,726 | 7,546 | 17,896 | 1,226 | 1.77 |
| *C. transvaalensis* | 28,444 | 25,260 | 3,184 | 16,595 | 345 | 1.52 |

**Supplementary Table 9. Positively selected genes.**

| **Groups** | **P-value** | **Gene** | **Function in Swiss-Prot** |
| --- | --- | --- | --- |
| ortholog22661 | 4.29E-10 | evm.model.LG09.1104 | CDK5RAP3-like protein |
| ortholog11385 | 4.73E-02 | evm.model.LG07.371 | Probable protein NAP1 |
| ortholog26835 | 1.31E-03 | evm.model.LG07.2271 | NA |
| ortholog00388 | 2.12E-02 | evm.model.LG06.1800 | Cytochrome P450 77A3 |
| ortholog26689 | 3.48E-03 | evm.model.LG07.1534 | Ultraviolet-B receptor UVR8 |
| ortholog14623 | 3.60E-02 | evm.model.LG02.1122 | Thioredoxin-like protein CDSP32, chloroplastic |
| ortholog01524 | 3.35E-03 | evm.model.LG05.731 | Signal recognition particle subunit SRP68 |
| ortholog18563 | 1.43E-02 | evm.model.LG03.2445 | NA |
| ortholog21116 | 6.13E-14 | evm.model.LG04.3371 | Pentatricopeptide repeat-containing protein At1g74900, mitochondrial |
| ortholog16320 | 1.67E-02 | evm.model.LG09.3402 | BTB/POZ domain-containing protein At1g04390 |
| ortholog02344 | 4.78E-02 | evm.model.LG02.1977 | 15-cis-phytoene desaturase, chloroplastic/chromoplastic |
| ortholog13328 | 3.11E-06 | evm.model.LG07.2383 | Putative kinase-like protein TMKL1 |
| ortholog18532 | 4.71E-08 | evm.model.LG03.2691 | NA |
| ortholog03746 | 4.71E-02 | evm.model.LG01.1791 | Zinc transporter 2 |
| ortholog09076 | 1.05E-02 | evm.model.LG04.3991 | Protein TIC 55, chloroplastic |
| ortholog03876 | 1.16E-02 | evm.model.LG02.2017 | NA |
| ortholog08684 | 8.15E-03 | evm.model.LG03.3570 | Pentatricopeptide repeat-containing protein At1g30610, chloroplastic |
| ortholog12968 | 1.33E-06 | evm.model.LG06.1177 | 2,3-dimethylmalate lyase |
| ortholog22735 | 3.31E-08 | evm.model.LG04.944 | L-gulonolactone oxidase 3 |
| ortholog24215 | 7.86E-03 | evm.model.LG09.1219 | Receptor like protein kinase S.2 |
| ortholog00803 | 3.35E-03 | evm.model.LG04.3218 | Probable inactive leucine-rich repeat receptor kinase XIAO |
| ortholog02704 | 6.88E-04 | evm.model.LG02.868 | UPF0235 protein At5g63440 |
| ortholog00037 | 1.05E-06 | evm.model.LG03.981 | Protein GIGANTEA |
| ortholog04749 | 7.51E-05 | evm.model.LG06.2231 | tRNA-dihydrouridine(47) synthase [NAD(P)(+)]-like |
| ortholog12895 | 2.06E-02 | evm.model.LG01.786 | Homocysteine S-methyltransferase 1 |
| ortholog24533 | 2.15E-03 | evm.model.LG03.4407 | Pentatricopeptide repeat-containing protein At5g39980, chloroplastic |
| ortholog15276 | 3.72E-08 | evm.model.LG09.1515 | Uncharacterized protein At2g34460, chloroplastic |
| ortholog16852 | 3.92E-02 | evm.model.LG09.1410 | Nitrile-specifier protein 5 |

**Supplementary Table 10. GO enrichment analyses in clusters for syntenic genes between zoysiagrass and bermudagrass.**

| **Cluster** | **ID** | **Description** | **P-value** |
| --- | --- | --- | --- |
| Cluster1 | GO:0003735 | structural constituent of ribosome | 1.31E-17 |
|  | GO:0006412 | translation | 2.75E-17 |
|  | GO:0005840 | ribosome | 3.36E-17 |
|  | GO:0005622 | intracellular | 8.50E-13 |
|  | GO:0003924 | GTPase activity | 8.32E-04 |
|  | GO:0004298 | threonine-type endopeptidase activity | 9.46E-04 |
|  | GO:0005839 | proteasome core complex | 9.46E-04 |
|  | GO:0051603 | proteolysis involved in cellular protein catabolic process | 9.46E-04 |
|  | GO:0005634 | nucleus | 1.39E-03 |
|  | GO:0005743 | mitochondrial inner membrane | 4.26E-03 |
|  | GO:0004175 | endopeptidase activity | 4.43E-03 |
|  | GO:0019773 | proteasome core complex alpha-subunit complex | 4.43E-03 |
|  | GO:0005525 | GTP binding | 4.48E-03 |
|  | GO:0000786 | nucleosome | 4.82E-03 |
|  | GO:0006334 | nucleosome assembly | 7.91E-03 |
|  | GO:0008017 | microtubule binding | 1.03E-02 |
|  | GO:0009725 | response to hormone | 1.09E-02 |
|  | GO:0010181 | FMN binding | 1.39E-02 |
|  | GO:0016757 | transferase activity transferring glycosyl groups | 1.50E-02 |
| Cluster2 | GO:0008237 | metallopeptidase activity | 1.68E-04 |
|  | GO:0009523 | photosystem II | 2.16E-03 |
|  | GO:0009690 | cytokinin metabolic process | 2.20E-03 |
|  | GO:0019139 | cytokinin dehydrogenase activity | 2.20E-03 |
|  | GO:0015979 | photosynthesis | 4.02E-03 |
|  | GO:0042393 | histone binding | 4.70E-03 |
|  | GO:0015079 | potassium ion transmembrane transporter activity | 5.13E-03 |
|  | GO:0071805 | potassium ion transmembrane transport | 5.13E-03 |
|  | GO:0004970 | ionotropic glutamate receptor activity | 6.18E-03 |
|  | GO:0006887 | exocytosis | 8.81E-03 |
|  | GO:0003824 | catalytic activity | 1.02E-02 |
| Cluster3 | GO:0003743 | translation initiation factor activity | 1.77E-03 |
|  | GO:0006413 | translational initiation | 2.58E-03 |
| Cluster4 | GO:0003700 | DNA-binding transcription factor activity | 1.67E-11 |
|  | GO:0003690 | double-stranded DNA binding | 3.17E-06 |
|  | GO:0043565 | sequence-specific DNA binding | 2.90E-05 |
|  | GO:0048544 | recognition of pollen | 5.14E-05 |
|  | GO:0022857 | transmembrane transporter activity | 4.31E-04 |

**Supplementary Table 11. GO enrichment of expanded genes.**

| **ID** | **Description** | **P-value** |
| --- | --- | --- |
| GO:0043531 | ADP binding | 1.00E-35 |
| GO:0016887 | ATPase activity | 5.81E-26 |
| GO:0042626 | ATPase activity coupled to transmembrane movement of substances | 4.12E-20 |
| GO:0005506 | iron ion binding | 6.79E-19 |
| GO:0016705 | oxidoreductase activity acting on paired donors with incorporation or reduction of molecular oxygen | 1.75E-18 |
| GO:0004252 | serine-type endopeptidase activity | 6.80E-13 |
| GO:0030247 | polysaccharide binding | 4.26E-12 |
| GO:0020037 | heme binding | 3.60E-11 |
| GO:0015079 | potassium ion transmembrane transporter activity | 4.85E-11 |
| GO:0071805 | potassium ion transmembrane transport | 4.85E-11 |
| GO:0006508 | proteolysis | 2.29E-09 |
| GO:0048544 | recognition of pollen | 6.59E-09 |
| GO:0004674 | protein serine/threonine kinase activity | 2.76E-07 |
| GO:0006633 | fatty acid biosynthetic process | 5.32E-07 |
| GO:0008234 | cysteine-type peptidase activity | 2.03E-06 |
| GO:0005092 | GDP-dissociation inhibitor activity | 2.70E-06 |
| GO:0007264 | small GTPase mediated signal transduction | 2.70E-06 |
| GO:0004556 | alpha-amylase activity | 6.70E-06 |
| GO:0005216 | ion channel activity | 1.02E-05 |
| GO:0000159 | protein phosphatase type 2A complex | 2.22E-05 |
| GO:0008272 | sulfate transport | 2.22E-05 |
| GO:0015116 | sulfate transmembrane transporter activity | 2.22E-05 |
| GO:0016747 | transferase activity transferring acyl groups other than amino-acyl groups | 2.29E-05 |
| GO:0022857 | transmembrane transporter activity | 2.35E-05 |
| GO:0017148 | negative regulation of translation | 2.37E-05 |
| GO:0030598 | rRNA N-glycosylase activity | 2.37E-05 |
| GO:0006811 | ion transport | 4.20E-05 |
| GO:0007275 | multicellular organism development | 6.30E-05 |
| GO:0008131 | primary amine oxidase activity | 7.31E-05 |
| GO:0009308 | amine metabolic process | 7.31E-05 |
| GO:0048038 | quinone binding | 7.31E-05 |
| GO:0016829 | lyase activity | 9.92E-05 |
| GO:0019888 | protein phosphatase regulator activity | 9.98E-05 |
| GO:0004970 | ionotropic glutamate receptor activity | 1.03E-04 |
| GO:0010333 | terpene synthase activity | 1.14E-04 |
| GO:0003993 | acid phosphatase activity | 1.67E-04 |
| GO:0000287 | magnesium ion binding | 7.80E-04 |
| GO:0008237 | metallopeptidase activity | 8.99E-04 |
| GO:0006511 | ubiquitin-dependent protein catabolic process | 1.10E-03 |
| GO:0004857 | enzyme inhibitor activity | 1.99E-03 |
| GO:0010181 | FMN binding | 2.01E-03 |
| GO:0004190 | aspartic-type endopeptidase activity | 2.23E-03 |
| GO:0005247 | voltage-gated chloride channel activity | 2.49E-03 |
| GO:0006821 | chloride transport | 2.49E-03 |
| GO:0016831 | carboxy-lyase activity | 2.73E-03 |
| GO:0030246 | carbohydrate binding | 3.30E-03 |
| GO:0019752 | carboxylic acid metabolic process | 4.42E-03 |

**Supplementary Table12. KEGG enrichment of expanded genes.**

| **ID** | **Description** | **P-value** |
| --- | --- | --- |
| ko00520 | Amino sugar and nucleotide sugar metabolism | 0.0014 |
| ko00052 | Galactose metabolism | 0.0276 |
| ko00380 | Tryptophan metabolism | 0.0276 |
| ko00010 | Glycolysis / Gluconeogenesis | 0.0403 |
| ko04016 | MAPK signaling pathway - plant | 0.0406 |
| ko00970 | Aminoacyl-tRNA biosynthesis | 0.0461 |

**Supplementary Table 13. Heat shock proteins (HSPs) in *C. transvaalensis.***

| **ID** | **Type** | **Function** |
| --- | --- | --- |
| evm.model.LG01.1038 | sHSP | 17.4 kDa class I heat shock protein OS=Oryza sativa subsp. japonica OX=39947 GN=HSP17.4 PE=2 SV=2 |
| evm.model.LG01.1036 | sHSP | 18.1 kDa class I heat shock protein OS=Oryza sativa subsp. japonica OX=39947 GN=HSP18.1 PE=2 SV=1 |
| evm.model.LG01.1039 | sHSP | 17.4 kDa class I heat shock protein OS=Oryza sativa subsp. japonica OX=39947 GN=HSP17.4 PE=2 SV=2 |
| evm.model.LG03.716 | sHSP | 16.9 kDa class I heat shock protein 1 OS=Oryza sativa subsp. japonica OX=39947 GN=HSP16.9A PE=1 SV=1 |
| evm.model.LG02.1296 | sHSP | 18.8 kDa class V heat shock protein OS=Oryza sativa subsp. japonica OX=39947 GN=HSP18.8 PE=2 SV=1 |
| evm.model.LG02.1517 | sHSP | 18.8 kDa class V heat shock protein OS=Oryza sativa subsp. japonica OX=39947 GN=HSP18.8 PE=2 SV=1 |
| evm.model.LG09.715 | sHSP | 26.2 kDa heat shock protein, mitochondrial OS=Oryza sativa subsp. japonica OX=39947 GN=HSP26.2 PE=2 SV=1 |
| evm.model.LG09.717 | sHSP | 26.2 kDa heat shock protein, mitochondrial OS=Oryza sativa subsp. japonica OX=39947 GN=HSP26.2 PE=2 SV=1 |
| evm.model.LG09.716 | sHSP | 26.2 kDa heat shock protein, mitochondrial OS=Oryza sativa subsp. japonica OX=39947 GN=HSP26.2 PE=2 SV=1 |
| evm.model.LG09.718 | sHSP | 26.2 kDa heat shock protein, mitochondrial OS=Oryza sativa subsp. japonica OX=39947 GN=HSP26.2 PE=2 SV=1 |
| evm.model.LG01.1276 | HSP40 | Chaperone protein DnaJ 1 OS=Thermus thermophilus (strain HB8 / ATCC 27634 / DSM 579) OX=300852 GN=dnaJ1 PE=3 SV=1 |
| evm.model.LG09.3282 | HSP40 | Chaperone protein dnaJ 8, chloroplastic OS=Arabidopsis thaliana OX=3702 GN=ATJ8 PE=2 SV=1 |
| evm.model.LG09.3281 | HSP40 | Chaperone protein dnaJ 8, chloroplastic OS=Arabidopsis thaliana OX=3702 GN=ATJ8 PE=2 SV=1 |
| evm.model.LG09.2754 | HSP40 | Chaperone protein dnaJ 10 OS=Arabidopsis thaliana OX=3702 GN=ATJ10 PE=2 SV=2 |
| evm.model.LG09.2797 | HSP40 | Chaperone protein dnaJ 10 OS=Arabidopsis thaliana OX=3702 GN=ATJ10 PE=2 SV=2 |
| evm.model.LG07.1935 | HSP40 | Chaperone protein dnaJ 10 OS=Arabidopsis thaliana OX=3702 GN=ATJ10 PE=2 SV=2 |
| evm.model.LG01.1243 | HSP40 | Chaperone protein DnaJ OS=Thermus thermophilus (strain HB27 / ATCC BAA-163 / DSM 7039) OX=262724 GN=dnaJ PE=3 SV=1 |
| evm.model.LG05.586 | HSP40 | DnaJ homolog subfamily B member 14 OS=Homo sapiens OX=9606 GN=DNAJB14 PE=1 SV=1 |
| evm.model.LG02.876 | HSP40 | DnaJ homolog subfamily B member 14 OS=Xenopus tropicalis OX=8364 GN=dnajb14 PE=2 SV=1 |
| evm.model.LG02.1036 | HSP40 | DnaJ homolog subfamily B member 8 OS=Mus musculus OX=10090 GN=Dnajb8 PE=2 SV=1 |
| evm.model.LG02.1098 | HSP40 | DnaJ homolog subfamily B member 8 OS=Mus musculus OX=10090 GN=Dnajb8 PE=2 SV=1 |
| evm.model.LG07.1341 | HSP40 | DnaJ homolog subfamily B member 9 OS=Rattus norvegicus OX=10116 GN=Dnajb9 PE=2 SV=2 |
| evm.model.LG07.1372 | HSP40 | DnaJ homolog subfamily B member 9 OS=Rattus norvegicus OX=10116 GN=Dnajb9 PE=2 SV=2 |
| evm.model.LG02.1162 | HSP40 | DnaJ homolog subfamily B member 9 OS=Rattus norvegicus OX=10116 GN=Dnajb9 PE=2 SV=2 |
| evm.model.LG07.2298 | HSP40 | DnaJ homolog subfamily C member 28 OS=Mus musculus OX=10090 GN=Dnajc28 PE=1 SV=2 |
| evm.model.LG07.2406 | HSP40 | DnaJ homolog subfamily C member 28 OS=Mus musculus OX=10090 GN=Dnajc28 PE=1 SV=2 |
| evm.model.LG08.1399 | HSP40 | DnaJ protein homolog 1 OS=Drosophila melanogaster OX=7227 GN=DnaJ-1 PE=1 SV=3 |
| evm.model.LG08.1415 | HSP40 | DnaJ protein homolog 1 OS=Drosophila melanogaster OX=7227 GN=DnaJ-1 PE=1 SV=3 |
| evm.model.LG09.169 | HSP60 | Chaperonin 60 subunit beta 1, chloroplastic OS=Arabidopsis thaliana OX=3702 GN=CPN60B1 PE=1 SV=3 |
| evm.model.LG06.1565 | HSP60 | Chaperonin 60 subunit beta 4, chloroplastic OS=Arabidopsis thaliana OX=3702 GN=CPN60B4 PE=1 SV=1 |
| evm.model.LG06.1608 | HSP60 | Chaperonin 60 subunit beta 4, chloroplastic OS=Arabidopsis thaliana OX=3702 GN=CPN60B4 PE=1 SV=1 |
| evm.model.LG08.10 | HSP70 | Heat shock 70 kDa protein 14 OS=Arabidopsis thaliana OX=3702 GN=HSP70-14 PE=1 SV=1 |
| evm.model.LG08.11 | HSP70 | Heat shock 70 kDa protein 14 OS=Arabidopsis thaliana OX=3702 GN=HSP70-14 PE=1 SV=1 |
| evm.model.LG08.254 | HSP70 | Heat shock 70 kDa protein 15 OS=Arabidopsis thaliana OX=3702 GN=HSP70-15 PE=1 SV=1 |
| evm.model.LG09.3422 | HSP70 | Heat shock 70 kDa protein 16 OS=Arabidopsis thaliana OX=3702 GN=HSP70-16 PE=2 SV=1 |
| evm.model.LG04.111 | HSP70 | Heat shock 70 kDa protein BIP1 OS=Oryza sativa subsp. japonica OX=39947 GN=BIP1 PE=1 SV=1 |
| evm.model.LG01.2524 | HSP70 | Heat shock 70 kDa protein BIP2 OS=Oryza sativa subsp. japonica OX=39947 GN=BIP2 PE=2 SV=1 |
| evm.model.LG03.2919 | HSP70 | Heat shock 70 kDa protein BIP5 OS=Oryza sativa subsp. japonica OX=39947 GN=BIP5 PE=1 SV=1 |
| evm.model.LG03.3009 | HSP70 | Heat shock 70 kDa protein BIP5 OS=Oryza sativa subsp. japonica OX=39947 GN=BIP5 PE=1 SV=1 |
| evm.model.LG06.1051 | HSP70 | Heat shock 70 kDa protein BIP5 OS=Oryza sativa subsp. japonica OX=39947 GN=BIP5 PE=1 SV=1 |
| evm.model.LG04.1264 | HSP70 | Heat shock 70 kDa protein, mitochondrial OS=Phaseolus vulgaris OX=3885 PE=2 SV=1 |
| evm.model.LG09.2482 | HSP70 | Heat shock 70 kDa protein, mitochondrial OS=Phaseolus vulgaris OX=3885 PE=2 SV=1 |
| evm.model.LG09.2733 | HSP70 | Heat shock 70 kDa protein, mitochondrial OS=Phaseolus vulgaris OX=3885 PE=2 SV=1 |
| evm.model.LG05.174 | HSP70 | Heat shock cognate 70 kDa protein OS=Petunia hybrida OX=4102 GN=HSP70 PE=2 SV=1 |
| evm.model.LG05.640 | HSP70 | Heat shock cognate 70 kDa protein OS=Petunia hybrida OX=4102 GN=HSP70 PE=2 SV=1 |
| evm.model.LG03.1873 | HSP70 | Heat shock cognate 70 kDa protein OS=Petunia hybrida OX=4102 GN=HSP70 PE=2 SV=1 |
| evm.model.LG03.4454 | HSP70 | Heat shock cognate 70 kDa protein OS=Petunia hybrida OX=4102 GN=HSP70 PE=2 SV=1 |
| evm.model.LG01.1086 | HSP70 | Heat shock cognate 70 kDa protein OS=Petunia hybrida OX=4102 GN=HSP70 PE=2 SV=1 |
| evm.model.LG01.1087 | HSP70 | Heat shock cognate 70 kDa protein OS=Petunia hybrida OX=4102 GN=HSP70 PE=2 SV=1 |
| evm.model.LG01.1088 | HSP70 | Heat shock cognate 70 kDa protein OS=Petunia hybrida OX=4102 GN=HSP70 PE=2 SV=1 |
| evm.model.LG01.1093 | HSP70 | Heat shock cognate 70 kDa protein OS=Petunia hybrida OX=4102 GN=HSP70 PE=2 SV=1 |
| evm.model.LG03.1693 | HSP70 | Stromal 70 kDa heat shock-related protein, chloroplastic OS=Pisum sativum OX=3888 GN=HSP70 PE=2 SV=1 |
| evm.model.LG08.917 | HSP70 | Stromal 70 kDa heat shock-related protein, chloroplastic OS=Pisum sativum OX=3888 GN=HSP70 PE=2 SV=1 |
| evm.model.LG08.940 | HSP70 | Stromal 70 kDa heat shock-related protein, chloroplastic OS=Pisum sativum OX=3888 GN=HSP70 PE=2 SV=1 |
| evm.model.LG04.1240 | HSP90 | Heat shock protein 81-1 OS=Oryza sativa subsp. japonica OX=39947 GN=HSP81-1 PE=3 SV=2 |
| evm.model.LG09.2686 | HSP90 | Heat shock protein 81-1 OS=Oryza sativa subsp. japonica OX=39947 GN=HSP81-1 PE=3 SV=2 |
| evm.model.LG07.1844 | HSP90 | Heat shock protein 81-1 OS=Oryza sativa subsp. japonica OX=39947 GN=HSP81-1 PE=3 SV=2 |
| evm.model.LG09.2689 | HSP90 | Heat shock protein 81-2 OS=Oryza sativa subsp. japonica OX=39947 GN=HSP81-2 PE=2 SV=1 |
| evm.model.LG06.2175 | HSP90 | Heat shock protein 90-1 OS=Arabidopsis thaliana OX=3702 GN=HSP90-1 PE=1 SV=3 |
| evm.model.LG09.2638 | HSP90 | Heat shock protein 90-5, chloroplastic OS=Arabidopsis thaliana OX=3702 GN=HSP90-5 PE=1 SV=1 |
| evm.model.LG07.1783 | HSP90 | Heat shock protein 90-5, chloroplastic OS=Arabidopsis thaliana OX=3702 GN=HSP90-5 PE=1 SV=1 |
| evm.model.LG03.2166 | HSP90 | Heat shock protein 90-6, mitochondrial OS=Arabidopsis thaliana OX=3702 GN=HSP90-6 PE=1 SV=1 |
| evm.model.LG04.2715 | HSP100 | ATP-dependent Clp protease ATP-binding subunit ClpC OS=Staphylococcus aureus (strain bovine RF122 / ET3-1) OX=273036 GN=clpC PE=1 SV=1 |
| evm.model.LG04.2714 | HSP100 | ATP-dependent Clp protease ATP-binding subunit ClpC OS=Staphylococcus aureus (strain MSSA476) OX=282459 GN=clpC PE=3 SV=1 |
| evm.model.LG04.1664 | HSP100 | ATP-dependent Clp protease ATP-binding subunit ClpC OS=Staphylococcus aureus (strain MW2) OX=196620 GN=clpC PE=3 SV=1 |
| evm.model.LG04.2710 | HSP100 | Probable ATP-dependent Clp protease ATP-binding subunit OS=Mycobacterium leprae (strain TN) OX=272631 GN=clpC PE=3 SV=2 |
| evm.model.LG04.2716 | HSP100 | Chaperone protein ClpB1 OS=Arabidopsis thaliana OX=3702 GN=CLPB1 PE=1 SV=2 |
| evm.model.LG03.3928 | HSP100 | Chaperone protein ClpB1 OS=Oryza sativa subsp. japonica OX=39947 GN=CLPB1 PE=2 SV=1 |
| evm.model.LG08.1907 | HSP100 | Chaperone protein ClpB1 OS=Oryza sativa subsp. japonica OX=39947 GN=CLPB1 PE=2 SV=1 |
| evm.model.LG01.1842 | HSP100 | Chaperone protein ClpB2, chloroplastic OS=Oryza sativa subsp. japonica OX=39947 GN=CLPB2 PE=2 SV=1 |
| evm.model.LG04.483 | HSP100 | Chaperone protein ClpB3, mitochondrial OS=Oryza sativa subsp. japonica OX=39947 GN=CLPB3 PE=2 SV=3 |
| evm.model.LG07.134 | HSP100 | Chaperone protein ClpC1, chloroplastic OS=Arabidopsis thaliana OX=3702 GN=CLPC1 PE=1 SV=1 |
| evm.model.LG06.1849 | HSP100 | Chaperone protein ClpC1, chloroplastic OS=Oryza sativa subsp. japonica OX=39947 GN=CLPC1 PE=2 SV=2 |
| evm.model.LG06.727 | HSP100 | Chaperone protein ClpC1, chloroplastic OS=Oryza sativa subsp. japonica OX=39947 GN=CLPC1 PE=2 SV=2 |
| evm.model.LG03.1658 | HSP100 | Chaperone protein ClpC2, chloroplastic OS=Oryza sativa subsp. japonica OX=39947 GN=CLPC2 PE=2 SV=2 |
| evm.model.LG07.199 | HSP100 | Chaperone protein ClpC3, chloroplastic OS=Oryza sativa subsp. japonica OX=39947 GN=CLPC3 PE=2 SV=1 |
| evm.model.LG07.300 | HSP100 | Chaperone protein ClpC4, chloroplastic OS=Oryza sativa subsp. japonica OX=39947 GN=CPLC4 PE=3 SV=1 |
| evm.model.LG07.55 | HSP100 | Chaperone protein ClpC4, chloroplastic OS=Oryza sativa subsp. japonica OX=39947 GN=CPLC4 PE=3 SV=1 |
| evm.model.LG04.2658 | HSP100 | Chaperone protein ClpD1, chloroplastic OS=Oryza sativa subsp. japonica OX=39947 GN=CLPD1 PE=2 SV=1 |
| evm.model.LG06.1810 | HSP100 | Chaperone protein ClpD2, chloroplastic OS=Oryza sativa subsp. japonica OX=39947 GN=CLPD2 PE=2 SV=2 |

**Supplementary Table 14. Total HSP70 family classifications among seven species.**

| **Gene Name** | **Species** | **Predicted Localization** | **Group** |
| --- | --- | --- | --- |
| AT1G09080 | *A. thaliana* | ER | I |
| AT1G11660 | *A. thaliana* | Nuclear | IV |
| AT1G16030 | *A. thaliana* | Cytoplasmic | V |
| AT1G56410 | *A. thaliana* | Cytoplasmic | V |
| AT1G79920 | *A. thaliana* | Cytoplasmic | V |
| AT1G79930 | *A. thaliana* | Cytoplasmic | V |
| AT2G32120 | *A. thaliana* | Cytoplasmic | V |
| AT3G09440 | *A. thaliana* | Cytoplasmic | V |
| AT3G12580 | *A. thaliana* | Cytoplasmic | V |
| AT4G16660 | *A. thaliana* | ER | I |
| AT4G24280 | *A. thaliana* | Chloroplast | II |
| AT4G37910 | *A. thaliana* | Mitochondrial | III |
| AT5G02490 | *A. thaliana* | Cytoplasmic | V |
| AT5G02500 | *A. thaliana* | Cytoplasmic | V |
| AT5G09590 | *A. thaliana* | Mitochondrial | III |
| AT5G28540 | *A. thaliana* | ER | I |
| AT5G42020 | *A. thaliana* | ER | I |
| AT5G49910 | *A. thaliana* | Chloroplast | II |
| BRADI_1g03720v3 | *B. distachyon* | Cytoplasmic | V |
| BRADI_1g32770v3 | *B. distachyon* | Nuclear | IV |
| BRADI_1g66470v3 | *B. distachyon* | Cytoplasmic | V |
| BRADI_1g66520v3 | *B. distachyon* | Cytoplasmic | V |
| BRADI_1g66527v3 | *B. distachyon* | Cytoplasmic | V |
| BRADI_1g66540v3 | *B. distachyon* | Cytoplasmic | V |
| BRADI_1g66550v3 | *B. distachyon* | Cytoplasmic | V |
| BRADI_1g66561v3 | *B. distachyon* | Cytoplasmic | V |
| BRADI_1g66590v3 | *B. distachyon* | Cytoplasmic | V |
| BRADI_1g69700v3 | *B. distachyon* | Cytoplasmic | V |
| BRADI_1g75681v3 | *B. distachyon* | Mix | VI |
| BRADI_1g77637v3 | *B. distachyon* | Mitochondrial | III |
| BRADI_2g06050v3 | *B. distachyon* | ER | I |
| BRADI_2g23250v3 | *B. distachyon* | Cytoplasmic | V |
| BRADI_2g30560v3 | *B. distachyon* | Chloroplast | II |
| BRADI_2g33676v3 | *B. distachyon* | Cytoplasmic | V |
| BRADI_2g33682v3 | *B. distachyon* | Cytoplasmic | V |
| BRADI_2g46937v3 | *B. distachyon* | Cytoplasmic | V |
| BRADI_2g54570v3 | *B. distachyon* | Cytoplasmic | V |
| BRADI_3g01477v3 | *B. distachyon* | ER | I |
| BRADI_3g53100v3 | *B. distachyon* | ER | I |
| BRADI_3g57450v3 | *B. distachyon* | Mitochondrial | III |
| BRADI_4g04220v3 | *B. distachyon* | Cytoplasmic | V |
| BRADI_4g28250v3 | *B. distachyon* | ER | I |
| BRADI_4g33878v3 | *B. distachyon* | Mitochondrial | III |
| BRADI_4g39470v3 | *B. distachyon* | Chloroplast | II |
| BRADI_4g39820v3 | *B. distachyon* | Mix | VI |
| BRADI_4g39850v3 | *B. distachyon* | Mix | VI |
| BRADI_4g43170v3 | *B. distachyon* | Mix | VI |
| BRADI_5g05900v3 | *B. distachyon* | Mitochondrial | III |
| evm.model.LG01.1084 | *C. transvaalensis* | Cytoplasmic | V |
| evm.model.LG01.1086 | *C. transvaalensis* | Cytoplasmic | V |
| evm.model.LG01.1087 | *C. transvaalensis* | Cytoplasmic | V |
| evm.model.LG01.1088 | *C. transvaalensis* | Cytoplasmic | V |
| evm.model.LG01.1089 | *C. transvaalensis* | Cytoplasmic | V |
| evm.model.LG01.1093 | *C. transvaalensis* | Cytoplasmic | V |
| evm.model.LG01.2524 | *C. transvaalensis* | ER | I |
| evm.model.LG01.778 | *C. transvaalensis* | Cytoplasmic | V |
| evm.model.LG02.83 | *C. transvaalensis* | Mix | VI |
| evm.model.LG02.87 | *C. transvaalensis* | Mix | VI |
| evm.model.LG03.1376 | *C. transvaalensis* | Mix | VI |
| evm.model.LG03.1693 | *C. transvaalensis* | Chloroplast | II |
| evm.model.LG03.1873 | *C. transvaalensis* | Cytoplasmic | V |
| evm.model.LG03.2338 | *C. transvaalensis* | Cytoplasmic | V |
| evm.model.LG03.2919 | *C. transvaalensis* | ER | I |
| evm.model.LG03.3009 | *C. transvaalensis* | ER | I |
| evm.model.LG03.4454 | *C. transvaalensis* | Cytoplasmic | V |
| evm.model.LG04.111 | *C. transvaalensis* | ER | I |
| evm.model.LG04.1264 | *C. transvaalensis* | Mitochondrial | III |
| evm.model.LG04.3543 | *C. transvaalensis* | ER | I |
| evm.model.LG05.174 | *C. transvaalensis* | Cytoplasmic | V |
| evm.model.LG05.640 | *C. transvaalensis* | Cytoplasmic | V |
| evm.model.LG06.1051 | *C. transvaalensis* | ER | I |
| evm.model.LG08.10 | *C. transvaalensis* | Cytoplasmic | V |
| evm.model.LG08.11 | *C. transvaalensis* | Cytoplasmic | V |
| evm.model.LG08.1550 | *C. transvaalensis* | Cytoplasmic | V |
| evm.model.LG08.254 | *C. transvaalensis* | Cytoplasmic | V |
| evm.model.LG08.917 | *C. transvaalensis* | Chloroplast | II |
| evm.model.LG08.940 | *C. transvaalensis* | Chloroplast | II |
| evm.model.LG09.2482 | *C. transvaalensis* | Mitochondrial | III |
| evm.model.LG09.2733 | *C. transvaalensis* | Mitochondrial | III |
| evm.model.LG09.3422 | *C. transvaalensis* | Nuclear | IV |
| Os01g08560 | *O. sativa* | Cytoplasmic | V |
| Os01g33360 | *O. sativa* | ER | I |
| Os01g49430 | *O. sativa* | Cytoplasmic | V |
| Os01g62290 | *O. sativa* | Cytoplasmic | V |
| Os02g02410 | *O. sativa* | ER | I |
| Os02g48110 | *O. sativa* | ER | I |
| Os02g53420 | *O. sativa* | Mitochondrial | III |
| Os03g02260 | *O. sativa* | Mitochondrial | III |
| Os03g11910 | *O. sativa* | Cytoplasmic | V |
| Os03g16860 | *O. sativa* | Cytoplasmic | V |
| Os03g16880 | *O. sativa* | Cytoplasmic | V |
| Os03g16920 | *O. sativa* | Cytoplasmic | V |
| Os03g50250 | *O. sativa* | ER | I |
| Os03g60620 | *O. sativa* | Cytoplasmic | V |
| Os05g08840 | *O. sativa* | Cytoplasmic | V |
| Os05g23740 | *O. sativa* | Chloroplast | II |
| Os05g30480 | *O. sativa* | ER | I |
| Os05g35400 | *O. sativa* | ER | I |
| Os05g38530 | *O. sativa* | Cytoplasmic | V |
| Os05g51360 | *O. sativa* | Mix | VI |
| Os06g10990 | *O. sativa* | Mix | VI |
| Os06g46600 | *O. sativa* | Nuclear | IV |
| Os08g09770 | *O. sativa* | ER | I |
| Os09g31486 | *O. sativa* | Mitochondrial | III |
| Os11g08440 | *O. sativa* | Cytoplasmic | V |
| Os11g08445 | *O. sativa* | Cytoplasmic | V |
| Os11g08460 | *O. sativa* | Cytoplasmic | V |
| Os11g08470 | *O. sativa* | Cytoplasmic | V |
| Os11g47760 | *O. sativa* | Cytoplasmic | V |
| Os12g05760 | *O. sativa* | Mix | VI |
| Os12g14070 | *O. sativa* | Chloroplast | II |
| Os12g38180 | *O. sativa* | Cytoplasmic | V |
| SEVIR_1G084600v2 | *S. viridis* | ER | I |
| SEVIR_1G116600v2 | *S. viridis* | ER | I |
| SEVIR_1G298600v2 | *S. viridis* | ER | I |
| SEVIR_2G007700v2 | *S. viridis* | Mix | VI |
| SEVIR_2G227500v2 | *S. viridis* | Mitochondrial | III |
| SEVIR_2G266700v2 | *S. viridis* | Mitochondrial | III |
| SEVIR_3G013500v2 | *S. viridis* | ER | I |
| SEVIR_3G050900v2 | *S. viridis* | Cytoplasmic | V |
| SEVIR_3G088800v2 | *S. viridis* | Chloroplast | II |
| SEVIR_3G108900v2 | *S. viridis* | Cytoplasmic | V |
| SEVIR_3G109000v2 | *S. viridis* | Cytoplasmic | V |
| SEVIR_3G222000v2 | *S. viridis* | Cytoplasmic | V |
| SEVIR_3G256300v2 | *S. viridis* | Chloroplast | II |
| SEVIR_3G338300v2 | *S. viridis* | Cytoplasmic | V |
| SEVIR_3G356800v2 | *S. viridis* | ER | I |
| SEVIR_4G058600v2 | *S. viridis* | Cytoplasmic | V |
| SEVIR_4G273900v2 | *S. viridis* | Nuclear | IV |
| SEVIR_5G188700v2 | *S. viridis* | ER | I |
| SEVIR_5G189700v2 | *S. viridis* | ER | I |
| SEVIR_5G381300v2 | *S. viridis* | Cytoplasmic | V |
| SEVIR_6G238700v2 | *S. viridis* | Mix | VI |
| SEVIR_7G335600v2 | *S. viridis* | Mix | VI |
| SEVIR_8G235800v2 | *S. viridis* | Cytoplasmic | V |
| SEVIR_9G032700v2 | *S. viridis* | Cytoplasmic | V |
| SEVIR_9G118900v2 | *S. viridis* | ER | I |
| SEVIR_9G190400v2 | *S. viridis* | Mitochondrial | III |
| SEVIR_9G455400v2 | *S. viridis* | Cytoplasmic | V |
| SEVIR_9G455800v2 | *S. viridis* | Cytoplasmic | V |
| SEVIR_9G492400v2 | *S. viridis* | Cytoplasmic | V |
| SORBI_3001G118600 | *S. bicolor* | ER | I |
| SORBI_3001G129000 | *S. bicolor* | Chloroplast | II |
| SORBI_3001G193500 | *S. bicolor* | Mitochondrial | III |
| SORBI_3001G418600 | *S. bicolor* | Cytoplasmic | V |
| SORBI_3001G419000 | *S. bicolor* | Cytoplasmic | V |
| SORBI_3001G419100 | *S. bicolor* | Cytoplasmic | V |
| SORBI_3001G419200 | *S. bicolor* | Cytoplasmic | V |
| SORBI_3001G419300 | *S. bicolor* | Cytoplasmic | V |
| SORBI_3001G419400 | *S. bicolor* | Cytoplasmic | V |
| SORBI_3001G419500 | *S. bicolor* | Cytoplasmic | V |
| SORBI_3001G419600 | *S. bicolor* | Cytoplasmic | V |
| SORBI_3001G419700 | *S. bicolor* | Cytoplasmic | V |
| SORBI_3001G419801 | *S. bicolor* | Cytoplasmic | V |
| SORBI_3001G419900 | *S. bicolor* | Cytoplasmic | V |
| SORBI_3001G420100 | *S. bicolor* | Cytoplasmic | V |
| SORBI_3001G454000 | *S. bicolor* | Cytoplasmic | V |
| SORBI_3002G008000 | *S. bicolor* | Mix | VI |
| SORBI_3002G249800 | *S. bicolor* | Mitochondrial | III |
| SORBI_3003G178101 | *S. bicolor* | ER | I |
| SORBI_3003G350700 | *S. bicolor* | Cytoplasmic | V |
| SORBI_3003G378700 | *S. bicolor* | ER | I |
| SORBI_3004G011700 | *S. bicolor* | ER | I |
| SORBI_3004G263500 | *S. bicolor* | ER | I |
| SORBI_3006G045200 | *S. bicolor* | ER | I |
| SORBI_3006G055600 | *S. bicolor* | Cytoplasmic | V |
| SORBI_3008G038700 | *S. bicolor* | Mix | VI |
| SORBI_3008G088200 | *S. bicolor* | Chloroplast | II |
| SORBI_3008G136000 | *S. bicolor* | Cytoplasmic | V |
| SORBI_3009G066900 | *S. bicolor* | Cytoplasmic | V |
| SORBI_3009G067000 | *S. bicolor* | Cytoplasmic | V |
| SORBI_3009G163900 | *S. bicolor* | Cytoplasmic | V |
| SORBI_3009G254800 | *S. bicolor* | Mix | VI |
| SORBI_3010G230600 | *S. bicolor* | Nuclear | IV |
| Zjn_sc00003.1.g04800.1.am.mk | *Z. japonica* | Cytoplasmic | V |
| Zjn_sc00007.1.g07070.1.am.mkhc | *Z. japonica* | ER | I |
| Zjn_sc00008.1.g02400.1.sm.mkhc | *Z. japonica* | Nuclear | IV |
| Zjn_sc00009.1.g07120.1.cf.mkhc | *Z. japonica* | Cytoplasmic | V |
| Zjn_sc00010.1.g03500.1.sm.mkhc | *Z. japonica* | Chloroplast | II |
| Zjn_sc00021.1.g00110.1.am.mk | *Z. japonica* | Cytoplasmic | V |
| Zjn_sc00022.1.g03460.1.sm.mkhc | *Z. japonica* | ER | I |
| Zjn_sc00024.1.g02420.1.sm.mkhc | *Z. japonica* | Cytoplasmic | V |
| Zjn_sc00024.1.g02430.1.am.mkhc | *Z. japonica* | Cytoplasmic | V |
| Zjn_sc00028.1.g02300.1.am.mk | *Z. japonica* | Cytoplasmic | V |
| Zjn_sc00038.1.g02610.1.cf.mkhc | *Z. japonica* | Cytoplasmic | V |
| Zjn_sc00040.1.g04780.1.cf.mkhc | *Z. japonica* | Cytoplasmic | V |
| Zjn_sc00047.1.g03790.1.sm.mkhc | *Z. japonica* | Mitochondrial | III |
| Zjn_sc00048.1.g00250.1.sm.mkhc | *Z. japonica* | Chloroplast | II |
| Zjn_sc00053.1.g00070.1.sm.mkhc | *Z. japonica* | Mitochondrial | III |
| Zjn_sc00075.1.g00100.1.sm.mkhc | *Z. japonica* | Chloroplast | II |
| Zjn_sc00128.1.g00850.1.cf.mkhc | *Z. japonica* | Cytoplasmic | V |
| Zjn_sc00160.1.g00320.1.sm.mk | *Z. japonica* | Mix | VI |
| Zjn_sc00160.1.g00460.1.am.mk | *Z. japonica* | Cytoplasmic | V |

**Supplementary Table 15. Orthologous gene pairs between *C. transvaalensis* and *Z. japonica***

| ***C. transvaalensis*** | ***Z. japonica*** |
| --- | --- |
| evm.model.LG03.1693 | Zjn_sc00010.1.g03500.1.sm.mkhc |
| evm.model.LG03.1693 | Zjn_sc00048.1.g00250.1.sm.mkhc |
| evm.model.LG03.1873 | Zjn_sc00038.1.g02610.1.cf.mkhc |
| evm.model.LG03.1873 | Zjn_sc00128.1.g00850.1.cf.mkhc |
| evm.model.LG03.2338 | Zjn_sc00038.1.g02610.1.cf.mkhc |
| evm.model.LG03.2338 | Zjn_sc00128.1.g00850.1.cf.mkhc |
| evm.model.LG03.4454 | Zjn_sc00009.1.g07120.1.cf.mkhc |
| evm.model.LG03.4454 | Zjn_sc00040.1.g04780.1.cf.mkhc |
| evm.model.LG04.1264 | Zjn_sc00047.1.g03790.1.sm.mkhc |
| evm.model.LG04.1264 | Zjn_sc00053.1.g00070.1.sm.mkhc |
| evm.model.LG04.3543 | Zjn_sc00007.1.g07070.1.am.mkhc |
| evm.model.LG04.3543 | Zjn_sc00022.1.g03460.1.sm.mkhc |
| evm.model.LG08.10 | Zjn_sc00024.1.g02420.1.sm.mkhc |
| evm.model.LG08.1550 | Zjn_sc00009.1.g07120.1.cf.mkhc |
| evm.model.LG08.1550 | Zjn_sc00040.1.g04780.1.cf.mkhc |
| evm.model.LG08.254 | Zjn_sc00024.1.g02420.1.sm.mkhc |
| evm.model.LG08.917 | Zjn_sc00075.1.g00100.1.sm.mkhc |
| evm.model.LG09.3422 | Zjn_sc00008.1.g02400.1.sm.mkhc |

**Supplementary Table 16. Tandem and segmental duplicated genes in *C. transvaalensis* and *Z. japonica***

|  | **Tandem duplicated gene pair** | **Segmental duplicated gene pair** |
| --- | --- | --- |
| ***C. transvaalensis*** | evm.model.LG01.1086:evm.model.LG01.1087 | evm.model.LG01.2524:evm.model.LG01.778 |
|  | evm.model.LG01.1087:evm.model.LG01.1088 | evm.model.LG01.778:evm.model.LG03.2919 |
|  | evm.model.LG01.1088:evm.model.LG01.1089 | evm.model.LG01.778:evm.model.LG03.3009 |
|  | evm.model.LG08.10:evm.model.LG08.11 | evm.model.LG01.1084:evm.model.LG06.1051 |
|  |  | evm.model.LG01.1084:evm.model.LG08.1550 |
|  |  | evm.model.LG01.2524:evm.model.LG06.1051 |
|  |  | evm.model.LG02.83:evm.model.LG05.640 |
|  |  | evm.model.LG03.1693:evm.model.LG05.640 |
|  |  | evm.model.LG03.1873:evm.model.LG03.2338 |
|  |  | evm.model.LG03.2338:evm.model.LG04.111 |
|  |  | evm.model.LG03.2919:evm.model.LG03.3009 |
|  |  | evm.model.LG03.2919:evm.model.LG06.1051 |
|  |  | evm.model.LG03.3009:evm.model.LG04.111 |
|  |  | evm.model.LG03.4454:evm.model.LG08.1550 |
|  |  | evm.model.LG04.111:evm.model.LG06.1051 |
|  |  | evm.model.LG04.3543:evm.model.LG09.2733 |
|  |  | evm.model.LG05.640:evm.model.LG06.1051 |
|  |  | evm.model.LG05.640:evm.model.LG08.254 |
|  |  | evm.model.LG06.1051:evm.model.LG08.917 |
|  |  | evm.model.LG08.10:evm.model.LG08.254 |
| ***Z. japonica*** | Zjn_sc00003.1.g04790.1.am.mk:Zjn_sc00003.1.g04800.1.am.mk | Zjn_sc00007.1.g07070.1.am.mkhc:Zjn_sc00022.1.g03460.1.sm.mkhc |
|  | Zjn_sc00024.1.g02420.1.sm.mkhc:Zjn_sc00024.1.g02430.1.am.mkhc | Zjn_sc00009.1.g07120.1.cf.mkhc:Zjn_sc00040.1.g04780.1.cf.mkhc |
|  |  | Zjn_sc00010.1.g03500.1.sm.mkhc:Zjn_sc00048.1.g00250.1.sm.mkhc |
|  |  | Zjn_sc00038.1.g02610.1.cf.mkhc:Zjn_sc00128.1.g00850.1.cf.mkhc |
|  |  | Zjn_sc00047.1.g03790.1.sm.mkhc:Zjn_sc00053.1.g00070.1.sm.mkhc |

**Supplementary Table 17. Fatty acid biosynthetic process gene families in *C. transvaalensis.***

| **ID** | **Function** |
| --- | --- |
| evm.model.LG04.1745 | 3-ketoacyl-CoA synthase 1 OS=Arabidopsis thaliana OX=3702 GN=KCS1 PE=1 SV=1 |
| evm.model.LG01.915 | 3-ketoacyl-CoA synthase 1 OS=Arabidopsis thaliana OX=3702 GN=KCS1 PE=1 SV=1 |
| evm.model.LG05.531 | 3-ketoacyl-CoA synthase 2 OS=Arabidopsis thaliana OX=3702 GN=KCS2 PE=2 SV=2 |
| evm.model.LG05.916 | 3-ketoacyl-CoA synthase 2 OS=Arabidopsis thaliana OX=3702 GN=KCS2 PE=2 SV=2 |
| evm.model.LG05.965 | 3-ketoacyl-CoA synthase 2 OS=Arabidopsis thaliana OX=3702 GN=KCS2 PE=2 SV=2 |
| evm.model.LG04.2146 | 3-ketoacyl-CoA synthase 2 OS=Arabidopsis thaliana OX=3702 GN=KCS2 PE=2 SV=2 |
| evm.model.LG08.2165 | 3-ketoacyl-CoA synthase 4 OS=Arabidopsis thaliana OX=3702 GN=KCS4 PE=2 SV=1 |
| evm.model.LG03.2923 | 3-ketoacyl-CoA synthase 5 OS=Arabidopsis thaliana OX=3702 GN=KCS5 PE=2 SV=1 |
| evm.model.LG04.3660 | 3-ketoacyl-CoA synthase 5 OS=Arabidopsis thaliana OX=3702 GN=KCS5 PE=2 SV=1 |
| evm.model.LG09.883 | 3-ketoacyl-CoA synthase 5 OS=Arabidopsis thaliana OX=3702 GN=KCS5 PE=2 SV=1 |
| evm.model.LG01.785 | 3-ketoacyl-CoA synthase 6 OS=Arabidopsis thaliana OX=3702 GN=CUT1 PE=1 SV=1 |
| evm.model.LG03.2920 | 3-ketoacyl-CoA synthase 6 OS=Arabidopsis thaliana OX=3702 GN=CUT1 PE=1 SV=1 |
| evm.model.LG03.2921 | 3-ketoacyl-CoA synthase 6 OS=Arabidopsis thaliana OX=3702 GN=CUT1 PE=1 SV=1 |
| evm.model.LG09.882 | 3-ketoacyl-CoA synthase 6 OS=Arabidopsis thaliana OX=3702 GN=CUT1 PE=1 SV=1 |
| evm.model.LG01.550 | 3-ketoacyl-CoA synthase 10 OS=Arabidopsis thaliana OX=3702 GN=FDH PE=1 SV=2 |
| evm.model.LG04.620 | 3-ketoacyl-CoA synthase 11 OS=Arabidopsis thaliana OX=3702 GN=KCS11 PE=1 SV=1 |
| evm.model.LG09.3076 | 3-ketoacyl-CoA synthase 11 OS=Arabidopsis thaliana OX=3702 GN=KCS11 PE=1 SV=1 |
| evm.model.LG01.439 | 3-ketoacyl-CoA synthase 11 OS=Arabidopsis thaliana OX=3702 GN=KCS11 PE=1 SV=1 |
| evm.model.LG08.2195 | 3-ketoacyl-CoA synthase 11 OS=Arabidopsis thaliana OX=3702 GN=KCS11 PE=1 SV=1 |

**Supplementary Table 18. KEGG enrichment of contracted genes.**

| **ID** | **Description** | **P-value** |
| --- | --- | --- |
| ko00940 | Phenylpropanoid biosynthesis | 7.7E-05 |
| ko03010 | Ribosome | 0.000917 |
| ko05418 | Fluid shear stress and atherosclerosis | 0.002588 |
| ko04075 | Plant hormone signal transduction | 0.004974 |
| ko00983 | Drug metabolism-other enzymes | 0.005509 |


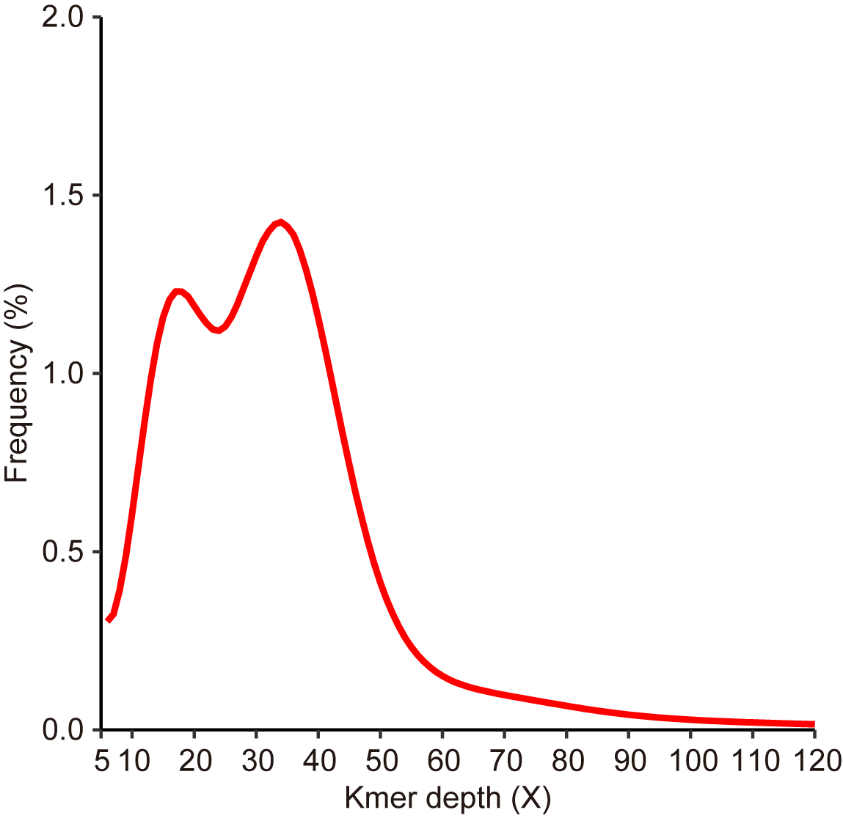


**Supplementary Fig. 1 *K*-mer distribution of Illumina reads for *C. transvaalensis.***


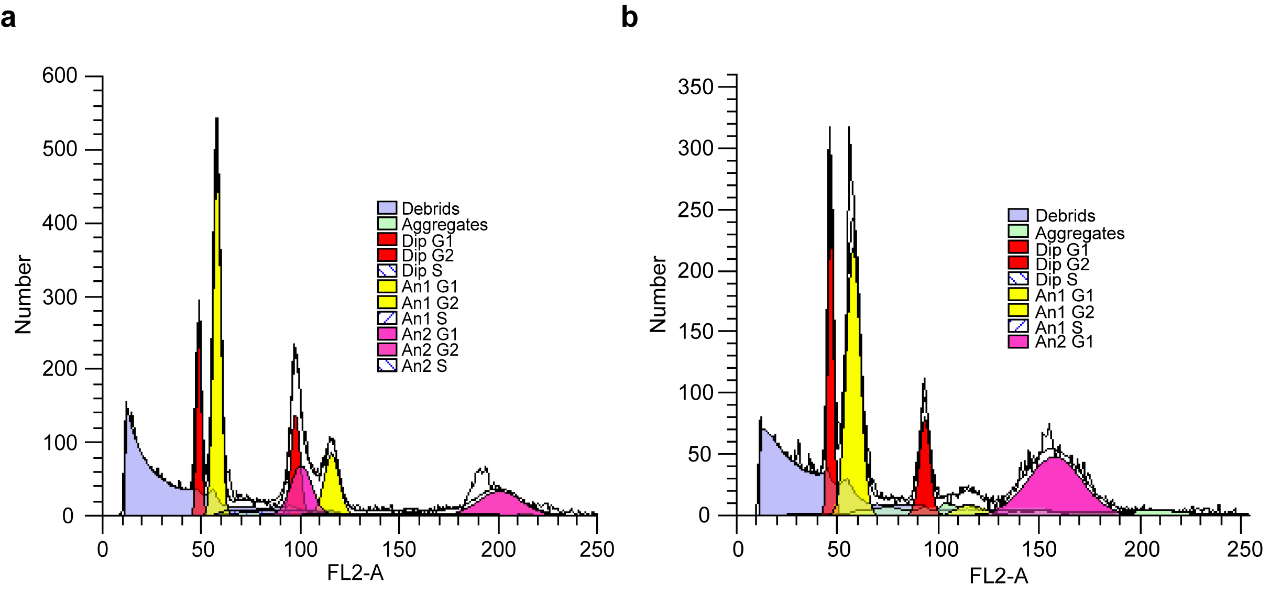


**Supplementary Fig. 2 Flow cytometry analyses of African bermudagrass (*C. transvaalensis*) and common bermudagrass (*C. dactylon*). a** Flow cytometry analysis of diploid African bermudagrass. *C. transvaalensis*, *S*. *bicolor* and *O*. *sativa* were yellow, pink and red, respectively. **b** Flow cytometry analysis of common bermudagrass. Common bermudagrass, *S*. *bicolor* and *Z*. *mays* were yellow, red and pink, respectively.


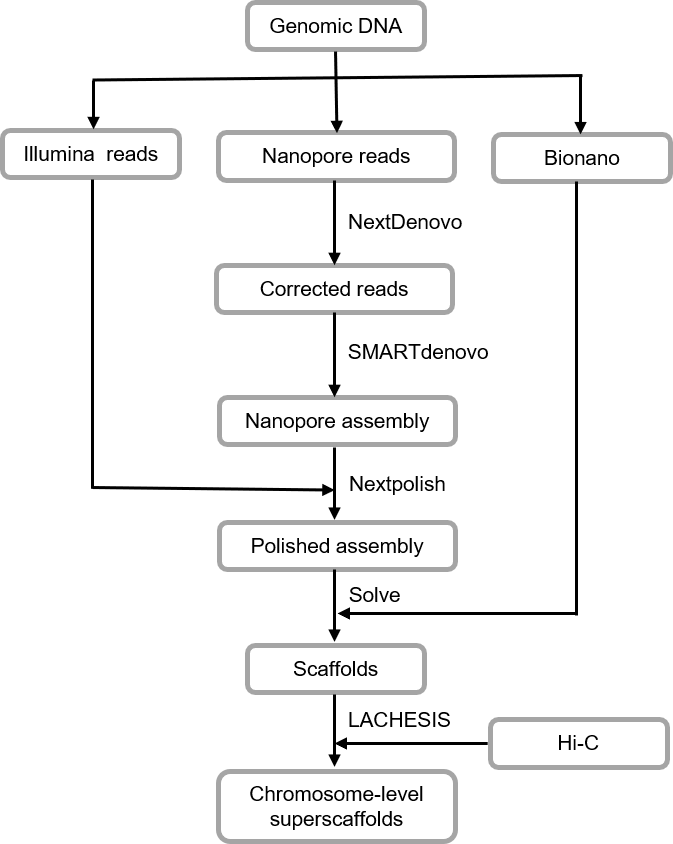


**Supplementary Fig. 3 The pipeline of genome assembly.**


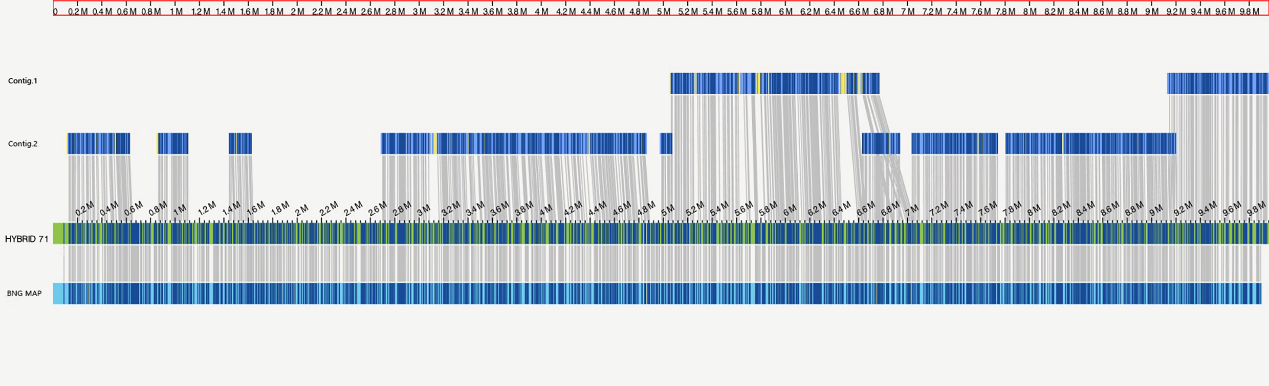


**Supplementary Fig. 4 Bionano optical map and contig linear relationship of a scaffold (scaffold_71).**


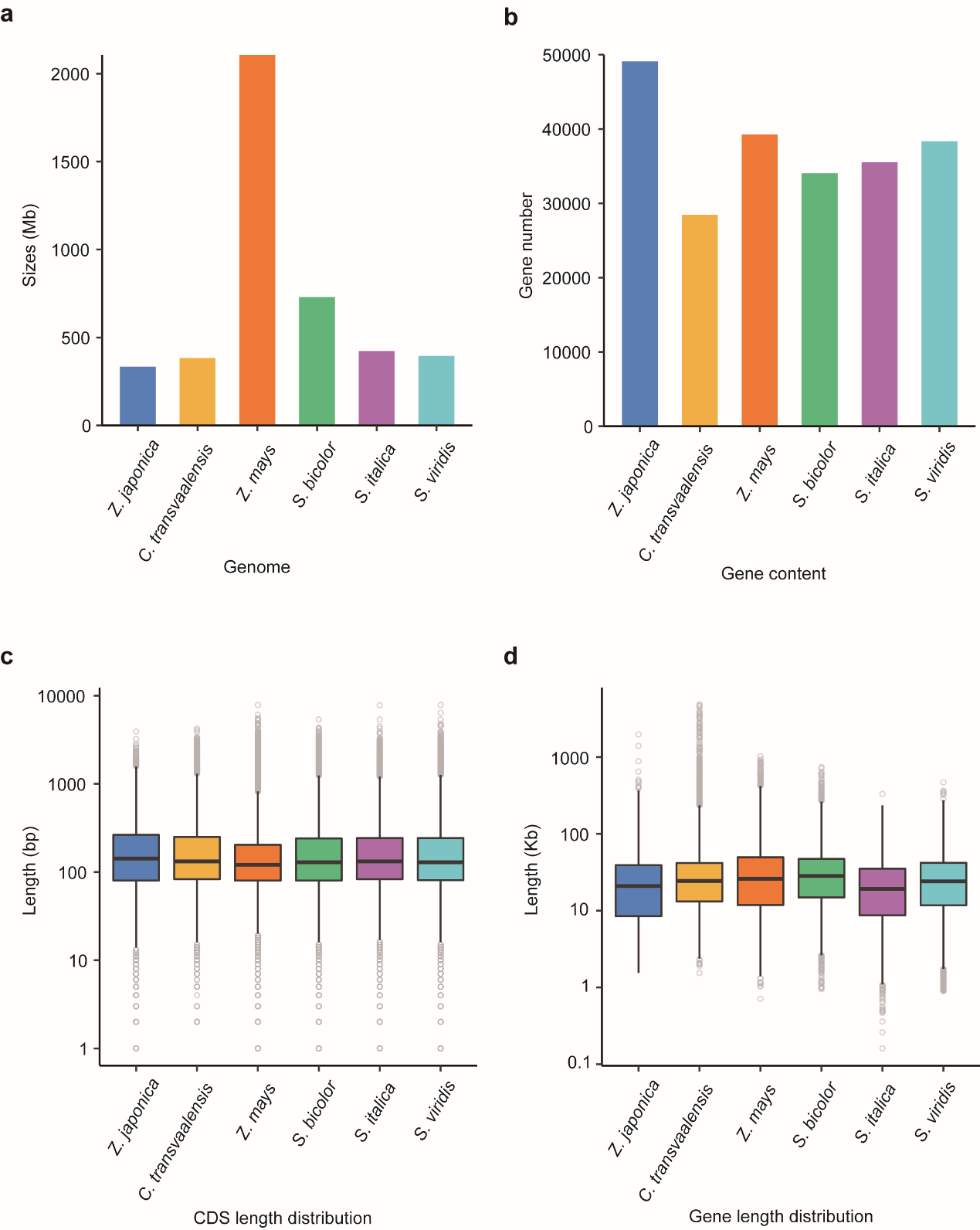


**Supplementary Fig. 5 Comparative analysis of the genome characteristics of *C. transvaalensis*, *Z*. *japonica*, *Z*. *mays*, *S*. *bicolor*, *S*. *italica* and *S*. *viridis***. **a** Comparison of genome size. **b** Comparison of gene number. **c** Comparison of coding sequences (CDS) length distribution. **d** Comparison of gene length distribution.


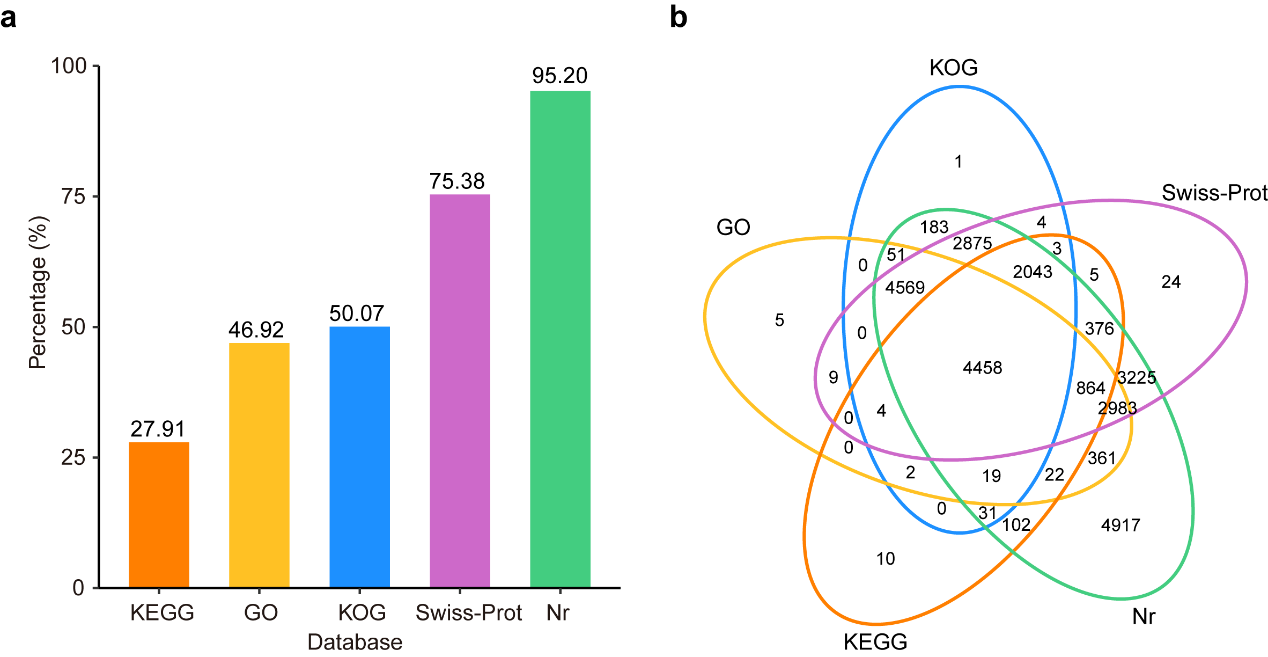


**Supplementary Fig. 6 Gene function annotation in databases (Swiss-Prot, Nr, KEGG, GO and KOG). a** Annotated gene function percentage across five databases. **b** Venn diagram of functionally annotated genes across five databases.


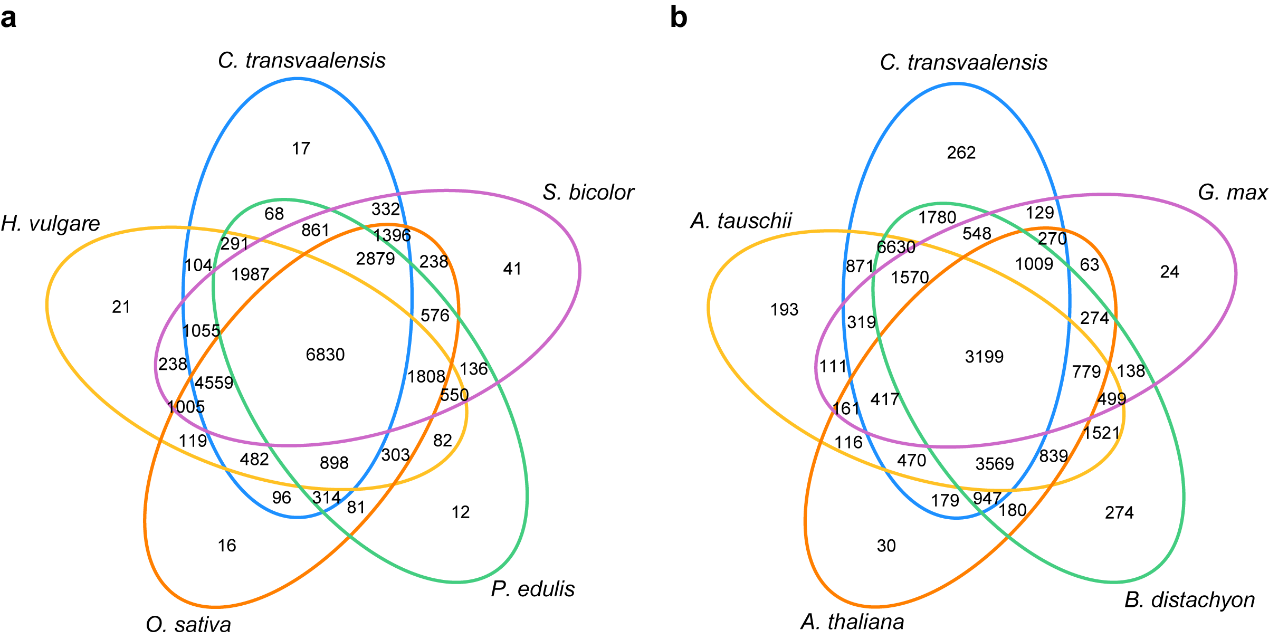


**Supplementary Fig. 7 Venn diagram of the number of shared gene families among *C. transvaalensis* and the other eight genomes (*A*. *tauschii*, *A*. *thaliana*, *B*. *distachyon*, *G*. *max*, *H*. *vulgare*, *O*. *sativa*, *P*. *edulis* and *S*. *bicolor*). a** The number of shared gene families among *C. transvaalensis*, *S*. *bicolor*, *H*. *vulgare*, *O*. *sativa* and *P*. *edulis*. **b** The number of shared gene families among *C. transvaalensis*, *A*. *tauschii*, *A*. *thaliana*, *B*. *distachyon* and *G*. *max*.


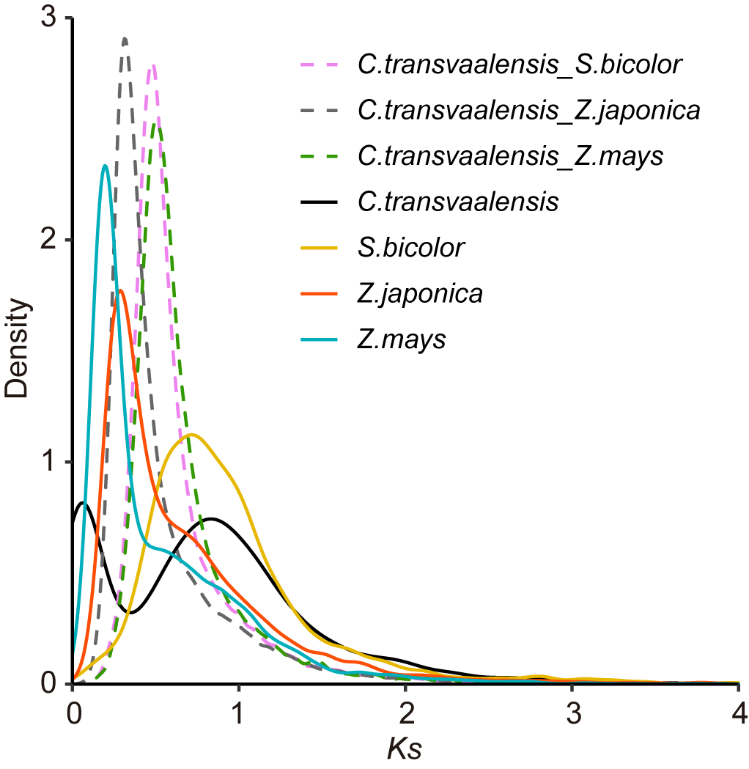


**Supplementary Fig. 8 Distribution of synonymous substitutions per site (*Ks*) for synteny genes within *C. transvaalensis*, *S*. *bicolor*, *Z*. *japonica* and *Z*. *mays.***


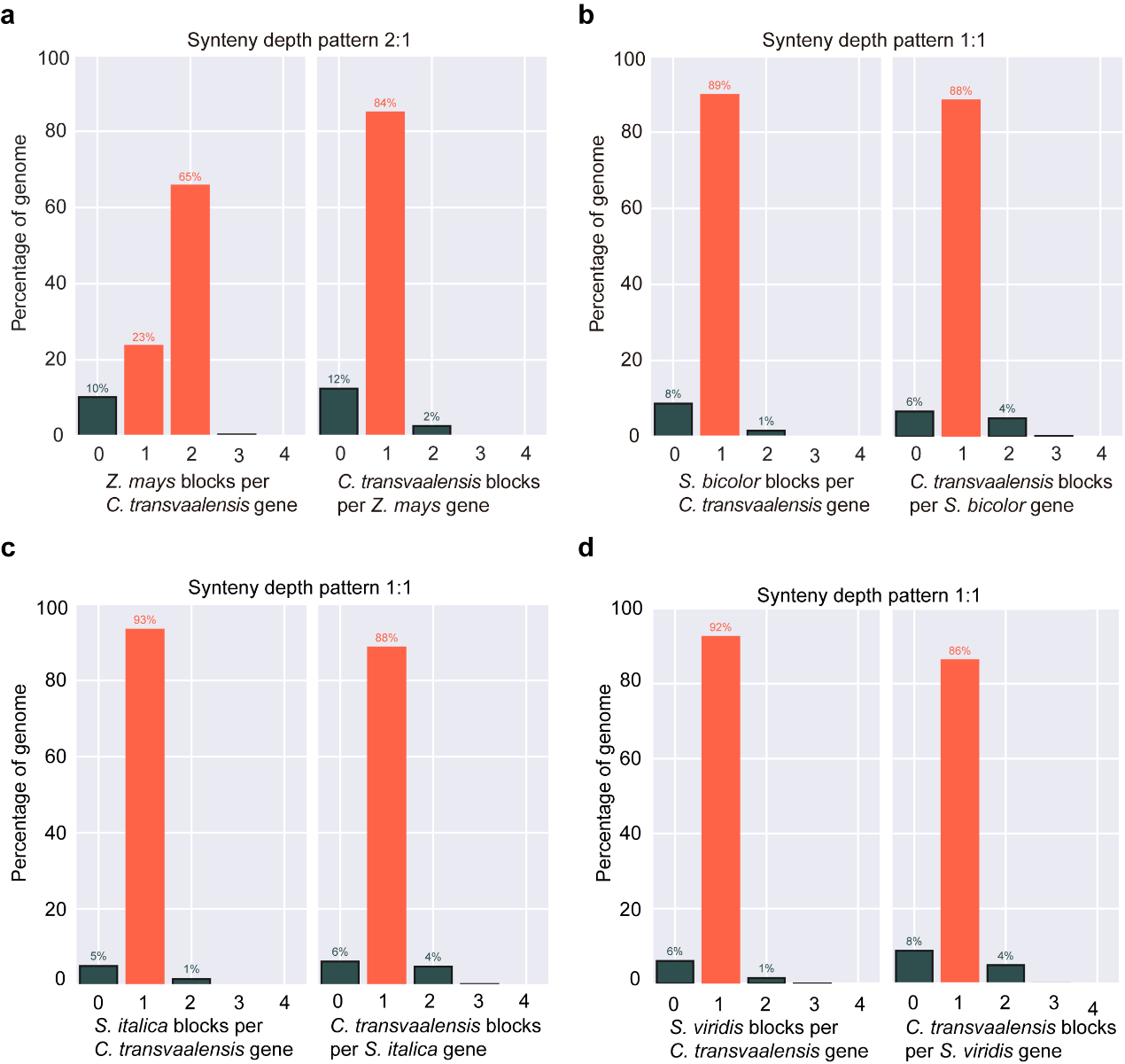

**Supplementary Fig. 9 Synteny depth patterns among *C. transvaalensis* vs. *Z*. *mays*, *C. transvaalensis* vs. *S. bicolor*, *C. transvaalensis* vs. *S. italica* and *C. transvaalensis* vs. *S. viridis.***


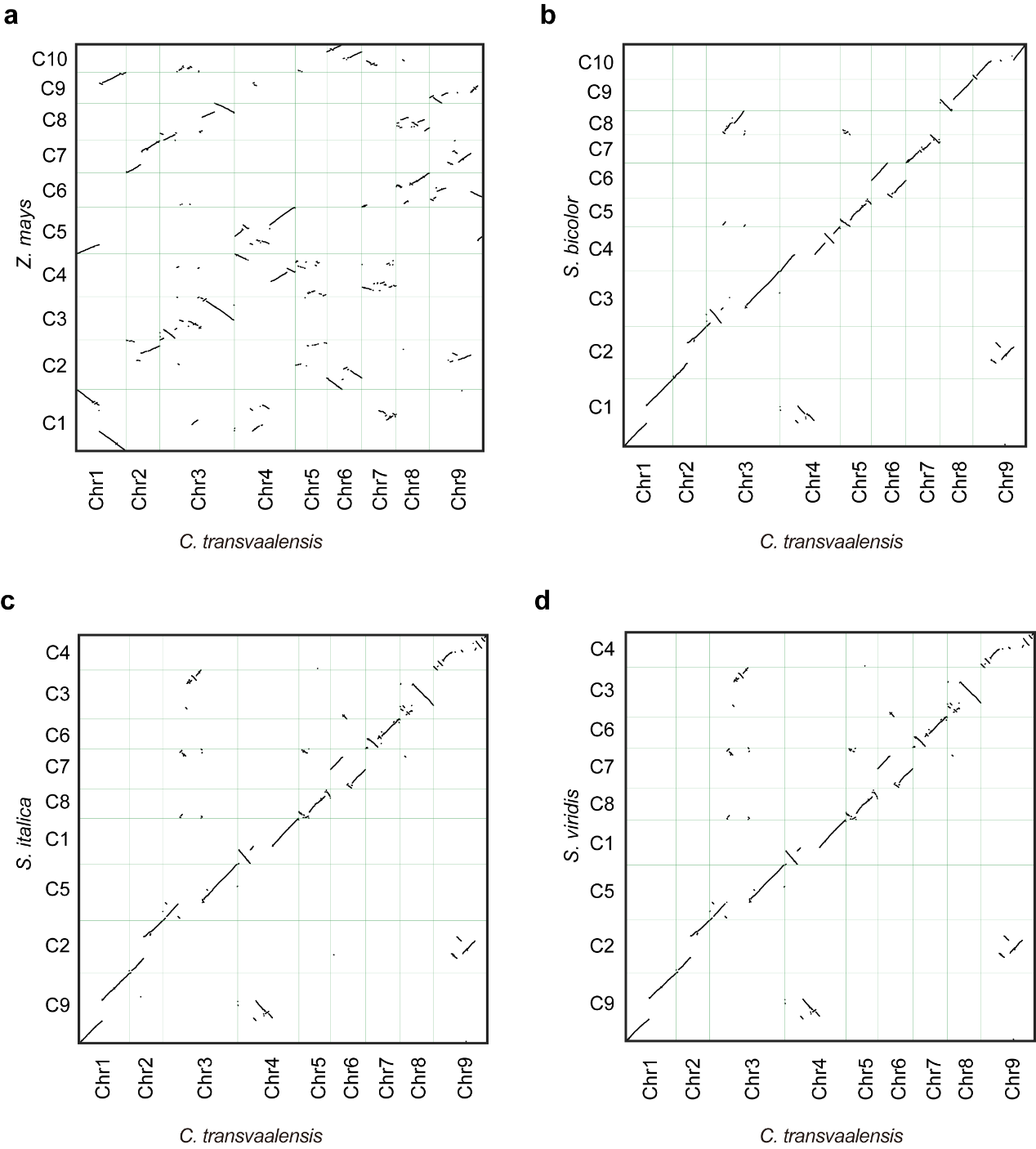


**Supplementary Fig. 10 Synteny dot plots among *C. transvaalensis* vs. *Z*. *mays*, *C. transvaalensis* vs. *S. bicolor*, *C. transvaalensis* vs. *S. italica* and *C. transvaalensis* vs. *S. viridis.***


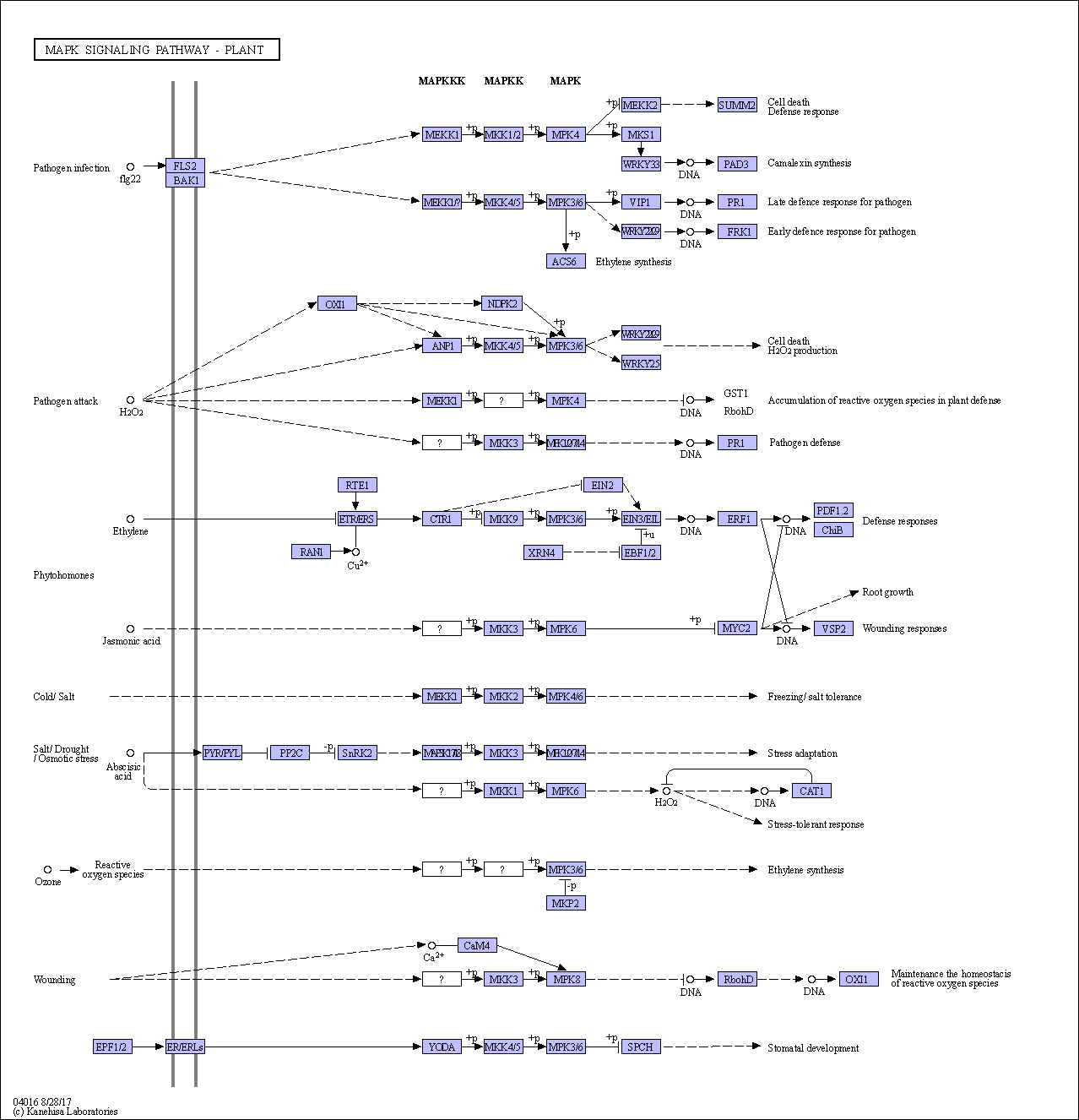


**Supplementary Fig. 11 MAPK signaling-plant pathway map.**


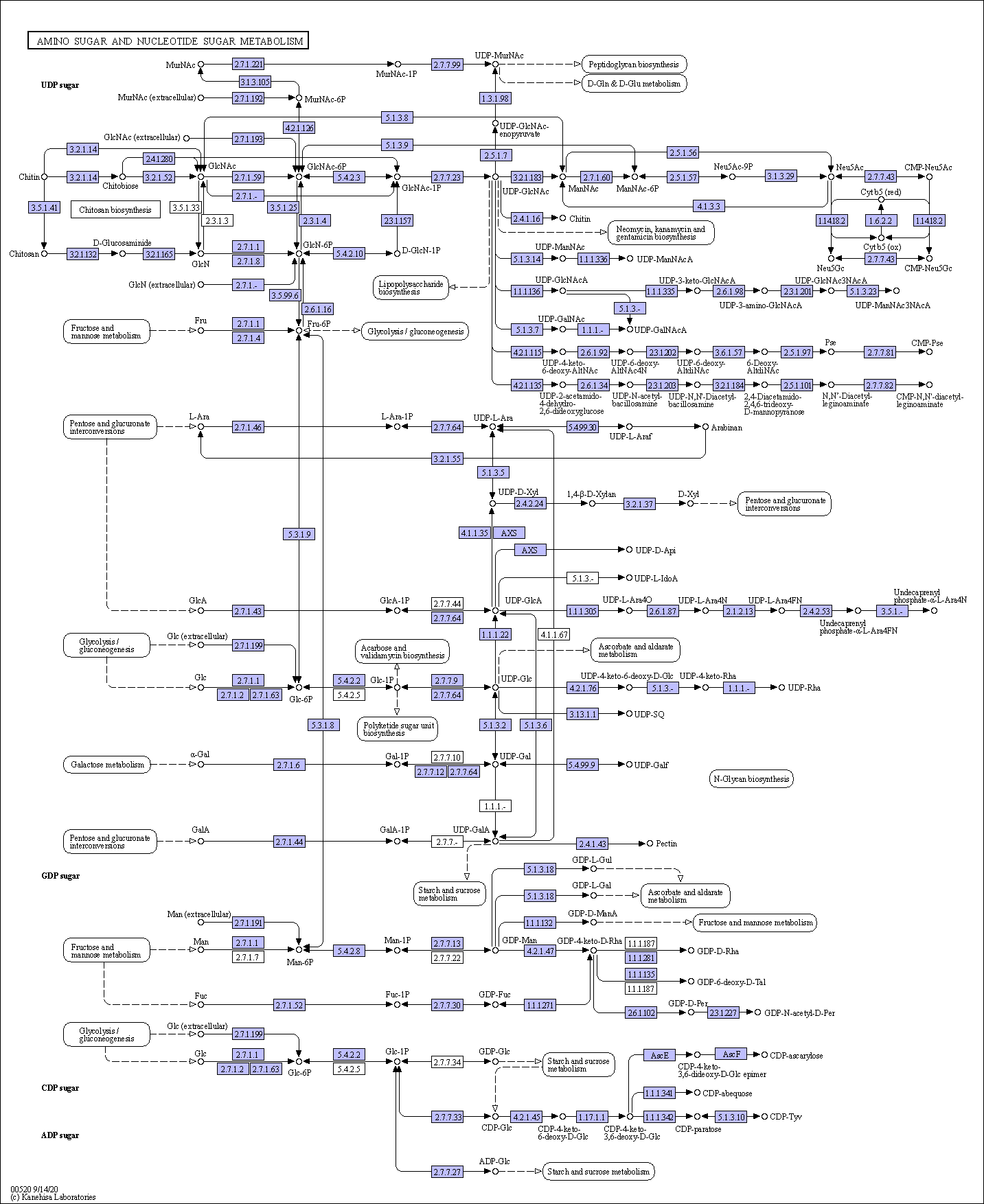


**Supplementary Fig. 12 Amino sugar and nucleotide sugar metabolism pathway map.**


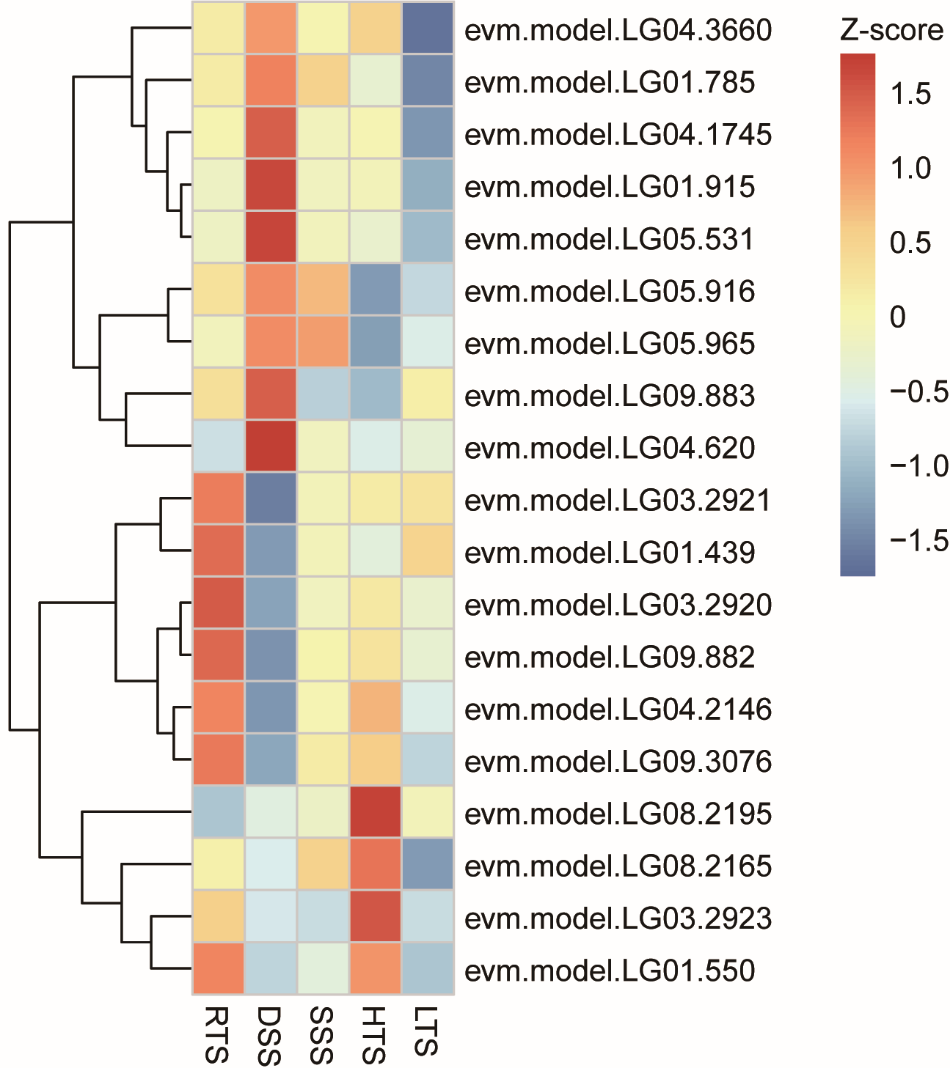


**Supplementary Fig. 13 Heatmap for expanded fatty acid biosynthetic process gene families.** RTS, HTS, DSS, SSS and LTS represent the shoot sample of *C. transvaalensis* under optimum temperature (25/30^o^C, day/night, control), high temperature (45^o^C for 6 h), drought stress (leaf relative water content of ~60%), salinity stress (200 mmol/L NaCl), and low temperature (4^o^C for 6 h), respectively.


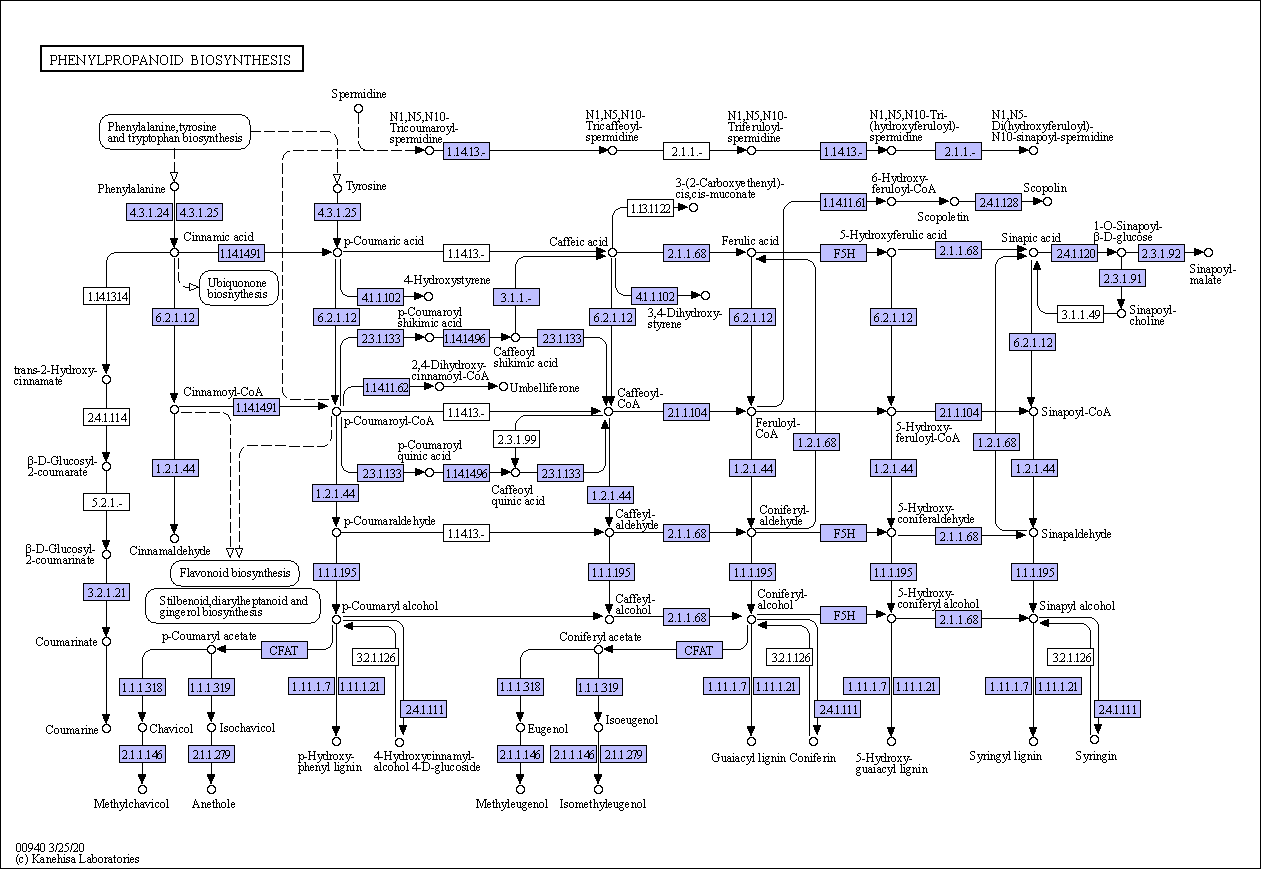


**Supplementary Fig. 14 Phenylpropanoid biosynthesis pathway map.**


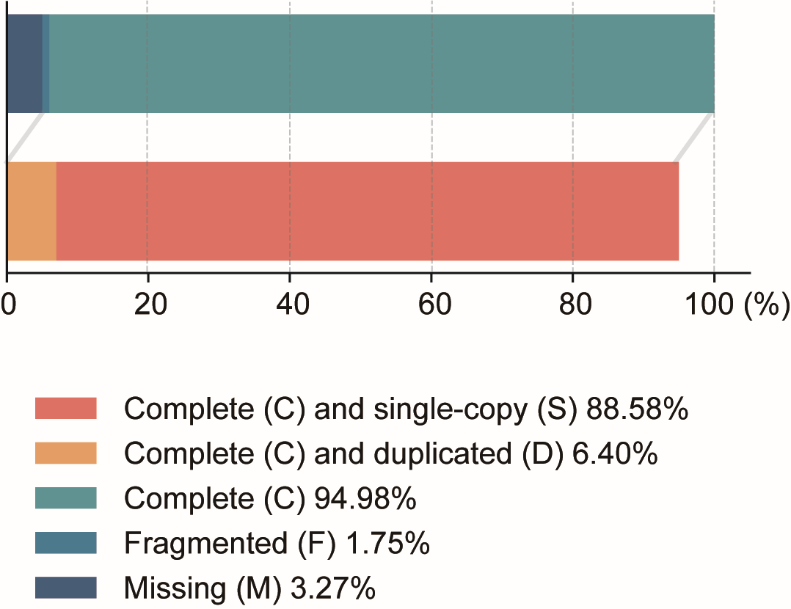


**Supplementary Fig. 15 BUSCO assessment of the annotated genes.**
